# Continuous Physics‐Informed Learning Expedited Battery Mechanism Decoupling

**DOI:** 10.1002/advs.202506772

**Published:** 2025-10-27

**Authors:** Shanling Ji, Jun Yuan, Bojing Zhang, Aleksei Sanin, Leon Merker, Zhisheng Zhang, Jianxiong Zhu, Helge Sören Stein

**Affiliations:** ^1^ School of Mechanical Engineering Southeast University Nanjing 211189 China; ^2^ TUM School of Natural Sciences Department of Chemistry Chair of Digital Catalysis Technical University of Munich (TUM) Lichtenbergstr. 4 85748 Garching Germany; ^3^ Munich Data Science Institute (MDSI) Technical University of Munich (TUM) Walther‐von‐Dyck‐Str. 10 85748 Garching Germany; ^4^ Munich Institute of Robotics and Machine Intelligence (MIRMI) Technical University of Munich (TUM) Georg‐Brauchle‐Ring 60 80992 Munich Germany; ^5^ Munich Institute of Integrated Materials Energy and Process (MEP) Technical University of Munich (TUM) Lichtenbergstr. 4a 85748 Garching Germany; ^6^ Munich Center for Machine Learning (MCML) Oettingenstraße 67 80538 Munich Germany

**Keywords:** aging prediction, battery model, mechanism diagnostics, physics‐informed machine learning

## Abstract

Accurate prediction of battery behavior under different dynamic operating conditions is critical for both fundamental research and practical applications. However, the diversity of emerging materials and cell architectures presents significant challenges to the generalizability of conventional prognostic approaches. Here, a novel physics‐informed battery modeling network (PIBMN) that integrates data‐driven learning with physical priors, enabling continuous parameter adaptation and broad applicability across cell formats and chemistries, is proposed. PIBMN effectively captures both fast and slow dynamic responses under a wide range of load profiles, applicable to both commercial and laboratory‐scale cells. By maintaining nonlinear expressivity while ensuring numerical stability, the model yields high‐fidelity, interpretable representations of internal electrochemical states. Beyond conventional health prognostics, PIBMN introduces a novel capability to decouple complex kinetics processes and concurrently track terminal voltage in real time, enabling mechanistic diagnostics with high resolution. As such, PIBMN establishes a versatile and scalable framework for in‐line quality control, adaptive cell‐specific battery management, and data‐informed optimization of next‐generation battery manufacturing processes.

## Graphical abstract

A battery modeling framework combining dual neural networks with physical prior is presented to track internal mechanisms, decouple overpotential components, and early predict aging trajectories using only DC data, eliminating the need for additional measurements. The proposed framework based on PINN and PIKAN is well aligned with the current drive toward explainable, data‐efficient AI models in energy systems.

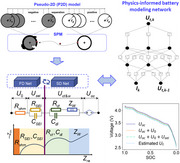

## 1 Introduction

Gradual performance degradation and opaque physicochemical states of batteries are major challenges in research and application.^[^ ^1^, ^2^, ^3^ ^]^ When these are, however, understood and monitored throughout the manufacturing and use phase, one could unlock more power and possibly extend the lifetime.^[^ ^4^, ^5^, ^6^, ^7^ ^]^ This necessitates high‐fidelity models such as pseudo‐2D representations in the Doyle Fuller Newman model (P2D‐DFN), which are great on short and intermediate time scales but require intimate knowledge about the internal cell chemistry and often presuppose opening a cell.^[^ ^8^ ^]^ Moreover, the setup complexity and specificity of P2D‐based models pose substantial barriers to their deployment across diverse chemistries, form factors, and operational conditions.^[^ ^9^ ^]^ This motivates a generalized, noninvasive parameter mapping approach that scales across scenarios.^[^ ^10^ ^]^ Beyond conventional aging prediction, a parameter estimation approach should robustly decouple fundamental electrochemical mechanisms using only readily accessible electrical (and thermal) signals.^[^ ^11^, ^12^, ^13^ ^]^

Transitioning from the laboratory environment to practical conditions with high‐rate cycling and nonlinear operations also demands operating batteries at non‐steady internal states.^[^ ^14^, ^15^ ^]^ The combinatorial chemistry and dynamics space, that is, the large variety of possible load conditions, in combination with the vast chemical space of cells, is virtually limitless. Recent research in machine learning (ML) for battery modeling has therefore tried to address this by developing models that either translate in time but are limited in protocol and chemistry,^[^ ^16^ ^]^ models that transfer across time but do not capture transient dynamics,^[^ ^17^, ^18^, ^19^ ^]^ and parameterization of physics models^[^ ^20^ ^]^ that translate to virtually any condition but not to another cell.^[^ ^21^, ^22^ ^]^ Moreover, all of these approaches need extensive data collection. We are, however, interested in training models from as little data as possible,^[^ ^23^ ^]^ while being able to utilize measurements from routine tests such as the galvanostatic intermittent titration technique (GITT) or formation process. As the terminal phase in battery production prior to leaving the factory, the formation process may serve as an additional quality control stage.^[^ ^24^ ^]^ Noninvasive parameter mapping, based on formation‐stage monitoring profiles, facilitates in‐line predictive defect detection to reduce the scrap rate and ensure durable performance.^[^ ^25^ ^]^

Physics‐informed machine learning (PIML) leverages mechanism‐based regularization and is well‐suited for small data regimes, effectively mitigating cross‐domain challenges associated with explainable representation.^[^ ^26^, ^27^, ^28^ ^]^ Such physics‐informed neural networks (PINNs) surrogate models are designed to estimate internal states otherwise only accessible by P2D and single‐particle models (SPM).^[^ ^29^ ^]^ Additionally, standard PINNs are built using multilayer perceptrons (MLPs), which enable automatic differentiation, but fixed activation functions may make it harder to learn the underlying physics. Compared to other approaches that integrate ML and mechanistic models, such as training ML models on physics‐generated data^[^ ^30^, ^31^, ^32^ ^]^ or using ML to optimize model parameters,^[^ ^8^, ^33^ ^]^ PINNs offer distinct advantages in ensuring physical consistency while requiring significantly less data.

Herein, we proposed a physics‐informed battery modeling network (PIBMN) incorporating dual temporal continual learning. We seek to strike a balance between high fidelity and low inference cost, with the benefit of being able to update the model on the fly at low computational cost. Instead of expanding datasets from singular operational regimes, the proposed methodology achieves cross‐domain applicability to industrial battery fabrication and electric vehicle dynamics through limited laboratory training samples. Besides a standard PINN with better traceability, we design a physics‐informed Kolmogorov–Arnold Network (PIKAN) to better explain and extract transient mechanisms through learned activation functions. The deployment on cells right after formation offers early aging prediction from formation alone. This paves the way for efficient deployment of automated formation grading, i.e., predicting potential use cases in electric vehicles, home storage, or grid stabilization prior to the factory gate. The PIBMN framework introduces a novel modeling paradigm that can be applied across cell formats and chemistries, integrating multi‐time scale physical prior into dual neural networks, thereby unlocking transient insights into mechanistic dynamics.

## 2 PIBMN Framework

**Figure 1:**
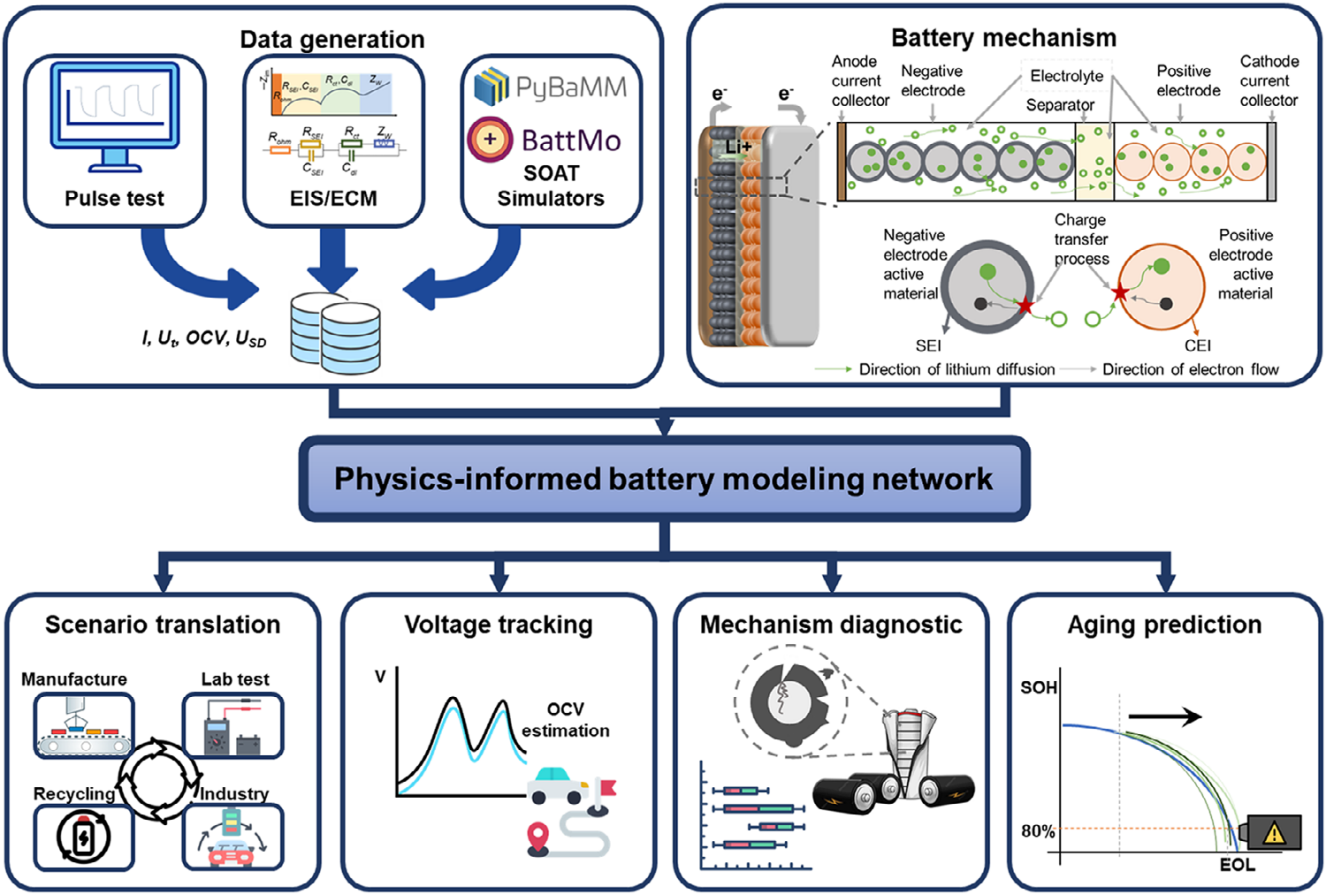
Schematic overview for multi‐time scale Li‐ion battery modeling. Data generation is for PIBMN training. P2D‐DFN is the electrochemical working mechanism. EEI is classified as solid‐electrolyte interphase (SEI) and cathode‐electrolyte interphase (CEI), which are formed on anode and cathode particle surfaces, respectively. The physical knowledge acquired from PIBMN can be used for scenario translation, voltage tracking, mechanism diagnostics, and aging prediction.

The proposed PIBMN approximates the parameter optimum via stitching the proposed governing equations (Note S1, Supporting Information) into the neural network training. As displayed in **Figure** **1**, the training set can be generated through various approaches that extract the open‐circuit voltage (OCV) and the voltage associated with slow dynamics. Advanced battery simulators like PyBaMM^[^ ^22^ ^]^ and BattMo^[^ ^21^ ^]^ offer valuable insights and detailed parameter guidance for this process. Additionally, pulse tests like GITT and EIS help refine parameter identification under real‐world application conditions. In common scenarios, electrical current and voltage signals can be fed into the ECM to pre‐estimate parameters for model training. The key contributions of PIBMN include scenario translation, voltage tracking, mechanism diagnostics, and aging prediction, providing a unique perspective to transmit electrical signals into underlying kinetics by physics‐constrained ML methods.

**Figure 2:**
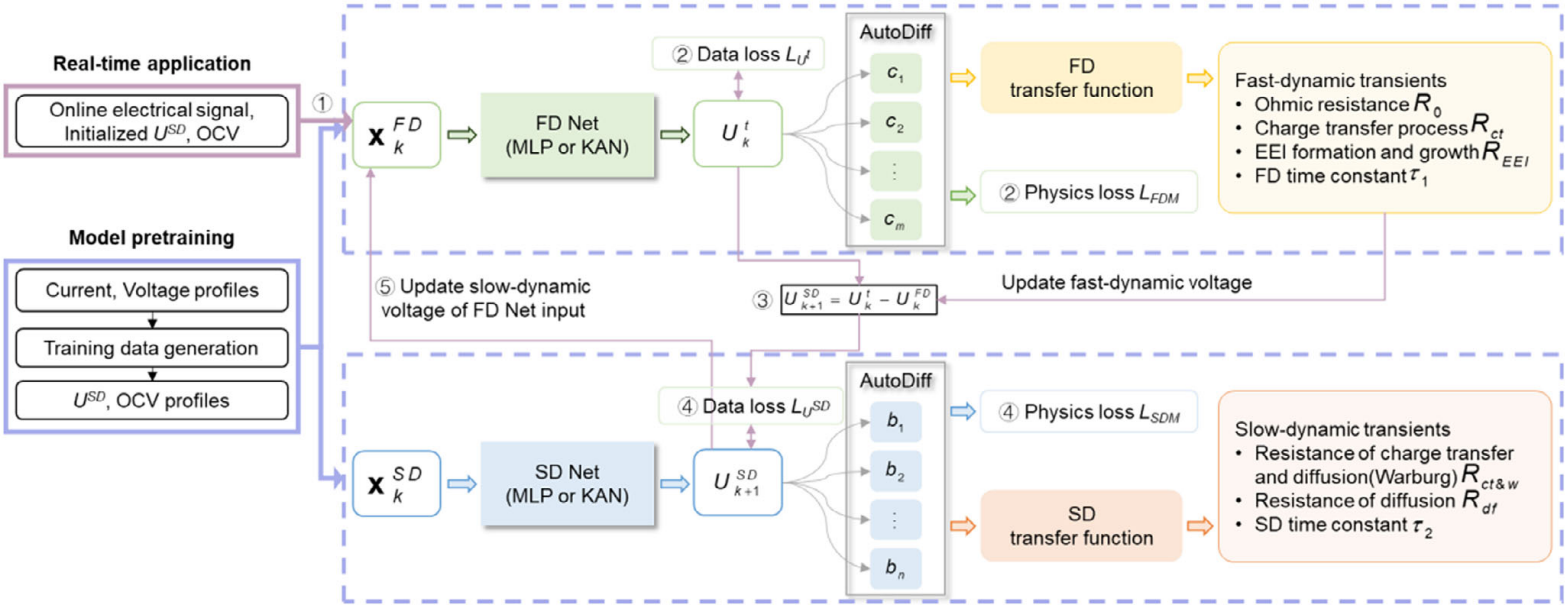
PIBMN architecture decouples fast‐slow dynamics according to integrated transfer functions. The FD Net comprises *m* input nodes and is set to 5 in this study. The SD Net comprises *n* input nodes and is set to 4 when simulating standard 2RC ECM and varies with different RC orders. *k* indicates the discrete time step.

The rocking‐chair‐like mechanism of Li‐ion batteries is shown schematically in Figure 1, highlighting the EEI, charge transfer through the interphase, and diffusion processes within the electrolyte and electrodes. Based on electrochemical impedance spectroscopy (EIS) analyses,^[^ ^34^ ^]^ charge transfer and lithium diffusion in two RC‐branch (2RC) ECM are herein treated in two tracks: fast dynamics (time constant < 10 s) and slow dynamics (time constant = 10–500 s). The PIBMN architecture, together with transfer functions of multi‐time scale ECM, is illustrated in **Figure** **2**, showing a fast‐dynamic network (FD Net) and a slow‐dynamic network (SD Net). Time constants are automatically estimated during continuous learning. In contrast to other parameterization methods,^[^ ^35^ ^]^ the “grey box” in the proposed framework is pre‐trained on transient data from a variety of cells, but prediction utilizes input data from a specific cell chemistry or dynamic load condition. In this study, while the PIBMN model is trained on certain cell chemistries and dynamic conditions of GITT and formation process, it is possible to predict a new unseen chemistry with a new unseen cycling condition, given an input cycle prior to the prediction cycle. This is a significant enhancement over existing methods, such as the deep learning model from Rahmanian et al.^[^ ^17^ ^]^ that also generalized over different chemistries but only predicted discharge capacity at a given C‐rate. The PIBMN has the ability to unveil transients while maintaining chemical generalization.

Dual neural networks are independently pretrained. Although the model input for real applications is only current and historical voltage, the training data needs additional reference profiles of slow‐dynamic voltage and OCV, which can be generated by solving 2RC ECM parameters or other methods in Figure 1. In this study, we combined the method of Dai et al.^[^ ^34^ ^]^ and Yang et al.^[^ ^36^ ^]^ to generate referable parameters. The parameter initialization during training data generation is set based on the cell type and previous related studies. Loss functions of the FD Net and the SD Net are described in Experimental Section “Loss Function.” Physics‐informed methods not only require less training data but also need fewer epochs (e.g., 10, 20, or 50) to converge compared to traditional deep learning.

During real‐time applications, model inputs are iteratively updated from online measurements and prior estimation. The procedure for the once update includes ①–⑤, as depicted in Figure 2. Different from the training process, there are important interactions between the FD Net and the SD Net to calculate data loss for the SD Net (step ③) and update the input of the FD Net (step ⑤). In step ③, the pseudo‐label for *U^SD^* is calculated by subtracting the estimated *U^FD^* from the measured terminal voltage *U^t^*. In step ⑤, the output of SD Net is fed back as an input to the FD Net, ensuring a continuous input stream for the latter. To explicitly enforce the distinction of time resolution between FD Net and SD Net, incorporating the distance between their estimated time constants into the loss function is recommended.

## 3 Results and Discussion

### 3.1 Voltage Tracking under Scenario Translation

**Figure 3:**
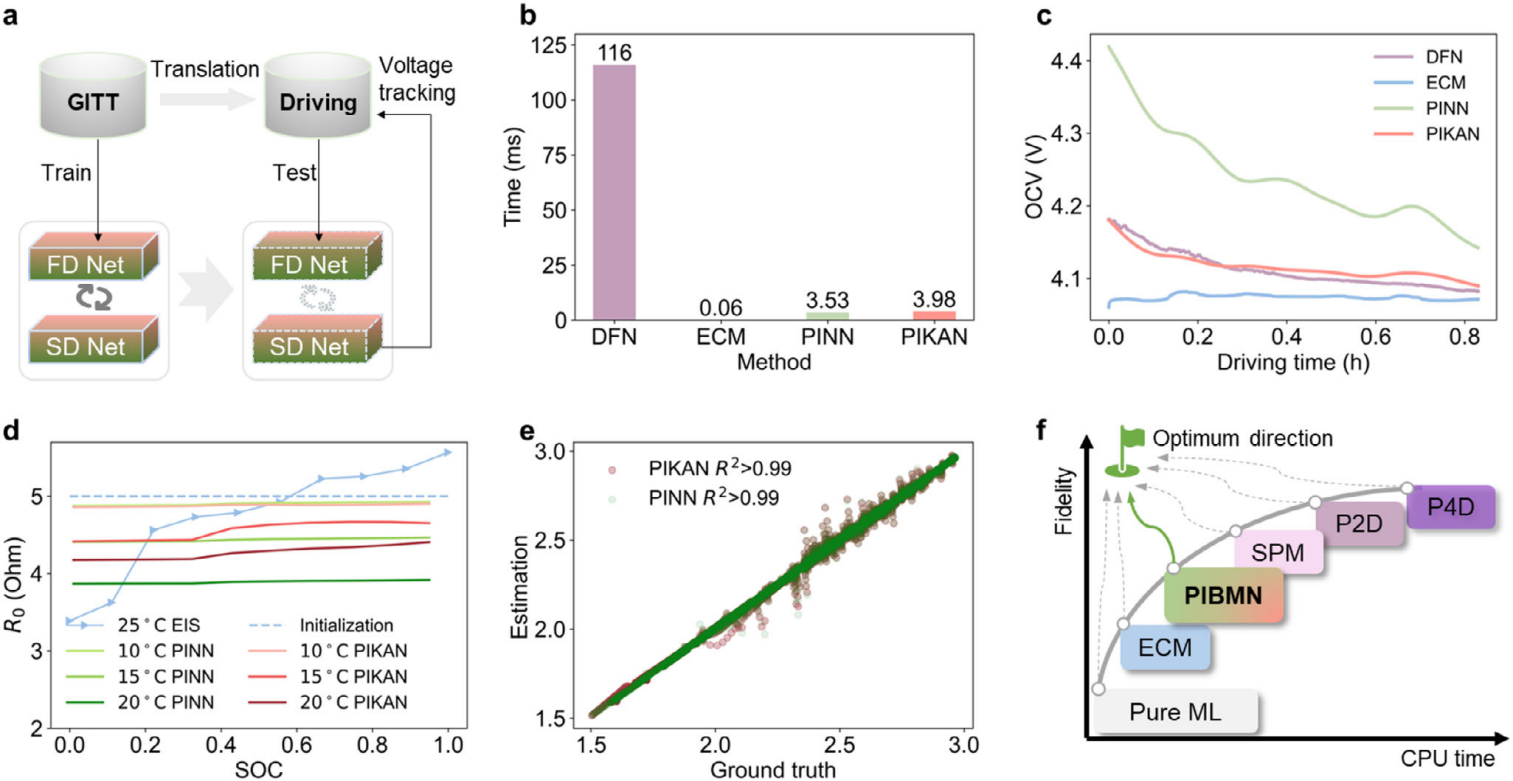
Performance across diverse scenarios. a) PIBMN implementation for translating from a GITT to a driving simulation. b) Comparison of wall time for voltage tracking (data is from PyBaMM simulation). c) OCV estimation under driving conditions (data is from PyBaMM simulation). The raw decoupled OCV curves are smoothed using Savitzky–Golay filters to mitigate current‐induced noise and clarify the presented trends. d) Estimated ohmic resistance under different temperatures (data is from Maxell ML2016 coin cells). e) Correlation between the estimated terminal voltage and ground truth (data is from Maxell ML2016 coin cells). For more data information, please refer to Experimental Section "Dataset Distribution." f) Comparison map of battery modeling methods.

Translating insights using pre‐trained models to drive applications is shown in **Figure** **3**a. Though extracting insights from a neural net can be challenging at times, as one is left with analyzing gradients and input influence.^[^ ^37^ ^]^ With the recent reintroduction of KAN, it is believed to be simpler to derive insights from not just weights and biases but also from a learned activation function on the edge of the network, that is, the extraction of the OCV and Warburg impedance. KAN linear^[^ ^38^ ^]^ is selected from improved KAN algorithms because it can be trained with torch tensors (see Experimental Section “MLP and KAN” for more information), and the PIKAN model is compared to PINN following the FAIR comparison principle.^[^ ^39^ ^]^ As shown in the comparison results in Figure 3b, two PIBMN models (PINN and PIKAN) accelerate the estimation of voltage transients compared to conventional DFN (simulated via PyBaMM^[^ ^22^ ^]^). Although still slower than 2RC ECM (solved via recursive least squares with Kalman filter^[^ ^36^ ^]^), the PIKAN model can estimate an accurate OCV profile that aligns with the ground truth (DFN) in Figure 3c. The R^2^ score of OCV estimation using the best‐trained PIKAN is 0.83. This more meticulous and precise estimation is delivered by learning nonlinear activation functions for PIKAN edges, but it also brings extra calculation burdens compared to PINN, because KAN is usually 10x slower than MLP, given the same parameter number.^[^ ^40^ ^]^ Besides, despite the nonlinear fitting capability of ECM solutions and the PIKAN method, their stability is low under steep input gradients. However, by reducing the network depth and using the robust optimizers (e.g., RAdam, Lookahead, AdamW^[^ ^41^ ^]^), these computational limitations of PIKAN can be addressed. The model configuration is described in Experimental Section “Training Configuration.” More details for the FAIR comparison between PIKAN and PINN are demonstrated in Note S2 (Supporting Information).

As depicted in Figure 3d,e, PINN and PIKAN are also employed on practical measured data under different ambient temperatures. The result implies an inverse relationship between battery internal resistance and temperature between 10 and 20 °C, consistent with most existing research. The SOC‐dependent internal resistance estimated via PIKAN shows more agreement with the uptrend characteristic behavior identified in EIS analyses (Figure 3d; Figure S1, Supporting Information). Besides, the nonlinear transformation of PINN with different fixed activation functions in Note S2 (Supporting Information), whereas the slope variation of extracted overpotentials cannot be related to electrochemical laws since it deviates from EIS observations. Despite that, the benefit of the voltage tracking ability of PINN is proven in Figure 3e, where the correlation between the estimated terminal voltage and ground truth is analyzed under driving test.

Conventional battery models, such as the P4D model in Figure 3f, achieve the highest fidelity level, integrating temperature dynamics, chemical reactions, and cell geometry, but come at high computational and setup costs. P4D and P2D models capture averaged microscopic variations in electrolyte concentration and electric potential along the thickness of a stack, with simplifications beyond that for single particles in SPM models. Conversely, overreliance on simplified computational methods like ECM or pure ML may result in low model fidelity. The auto‐regressive with extra inputs (ARX) model,^[^ ^42^ ^]^ derived from the ECM or fractional‐order model, faces challenges related to initialization and convergence. Pure ML‐based parameter mapping by narrowing the gap between the latent feature distribution of input and output is an alternate approach, but sometimes needs thousands of simulations and is not facile to update. To this end, PIBMN could be the optimum approach for battery modeling that balances CPU computation time and model fidelity.^[^ ^10^, ^43^, ^44^ ^]^

### 3.2 Extension to Higher‐Fidelity Battery Modeling

**Figure 4:**
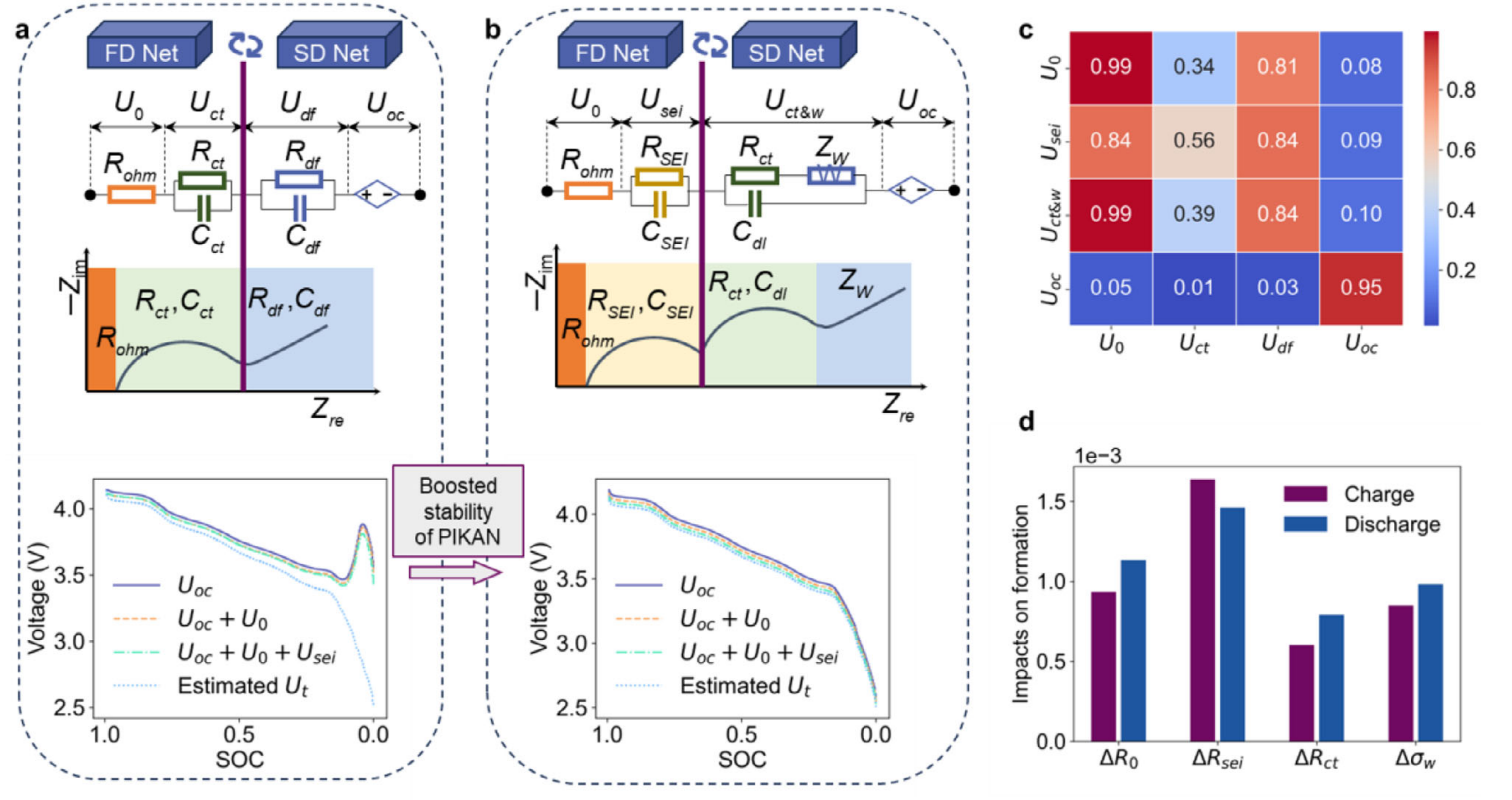
Comparison of two ECMs. a) PIKAN with 2RC ECM. b) PIKAN with the higher‐fidelity ECM model. c) Spearman coefficients between two kinds of voltage decomposition. d) Absolute SHAP values of resistance variations on formation performance.

The PIBC above mainly emulates two underlying kinetics with RC branches. However, a high‐order model may provide more electrochemical details, such as SEI layer growth and diffusion coefficients. As displayed in **Figure** **4**a,b, the extended model considers a Warburg impedance and SEI layer. Although the FD Net cannot recognize the dynamic process of SEI or CEI, only SEI is considered in the model because it accounts for a larger proportion of the formation process. The related higher‐fidelity AFX model is derived and shown in Note S3 (Supporting Information). The Warburg element transfer equations enable SD Net to integrate extended historical dependencies, thereby enhancing computational stability in PIKAN implementations. Notably, during the terminal discharge phase, where voltage exhibits a stark decline, the refined model achieves improved voltage curve smoothness through recursive integration of multi‐step historical states. Furthermore, architectural simplification via reduced KAN layers combined with gradient norm constraints effectively mitigates gradient vanishing or gradient explosion risks. The decomposed driving cycle based on the improved model is displayed in Figure S2 (Supporting Information), illustrating an interesting possibility of monitoring SEI resistance for vehicle batteries.

Spearman coefficients are calculated to analyze the correlation among voltage components of the two models, as shown in Figure 4c. The rank‐ordering Spearman statistic is chosen because it is robust to nonlinear relationships among branch voltages (compared to Pearson coefficients for the same ECM, shown in Figure S3, Supporting Information). Estimated ohmic overpotential (*U*_0_) and OCV (*U*_oc_) from the two models suggest a strong relationship with a coefficient approaching 0.95. The map also alludes *U_sei_* is related to *U*_0_, *U_ct_*, and *U_df_*. The coefficient associated with *U_ct_* is relatively low, potentially reflecting differences in the accuracy of simulating the charge transfer process between the two methods. Faster implementation obtained through PINN is shown in Figure S4 (Supporting Information) and demonstrates inferior explainability with negative ohmic resistance estimation, compared to PIKAN results in Figure S5 (Supporting Information).

The initial charge and discharge cycle, also known as the formation, primarily forms the SEI layer inside batteries and therefore affects lifetime. Figure 4d displays the SHAP importance of resistance variations in relation to battery formation performance, which includes capacity loss and coulombic efficiency. The difference in SEI resistance of the first two cycles shows the most obvious impact on the formation results. The SEI resistance distribution across the four formation processes (Figure S6, Supporting Information) further reveals significant changes in the SEI after the initial two formation cycles. Hence, continuous assessment of SEI formation dynamics using the proposed analytical framework is strongly advised to enable proactive battery management strategies.

Notably, all degradation cycling data presented in this study are acquired under temperature‐controlled constant current charging‐discharging (CCCD) protocols, ensuring experimental repeatability and condition uniformity across the dataset. Typically, it is suggested that the diffusion and charge transfer processes are quantified under steady‐state conditions, such as impedance or pulse tests.^[^ ^45^ ^]^ At the same time, since large current densities can exacerbate the polarization of internal chemical systems, most studies are dedicated to incremental capacity analyses (ICA) to detect phase transition and quantify battery aging based on small current rates (≤ 1C). ICA by decomposed overpotential is supplied in Note S4 (Supporting Information).

### 3.3 Unlocking Degradation Mechanisms for Formation Optimization

**Figure 5:**
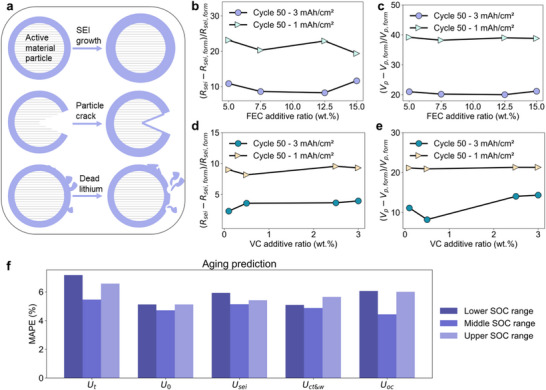
Demonstration of mechanisms decoupling during formation and aging. a) Schematic diagram of SEI growth, particle cracking, and dead lithium. b) and c) are SEI growth and polarization voltage for different FEC additive ratios. d) and e) are investigations on varying VC additive ratios. f) Aging prediction comparison of different voltage components.

Understanding aging mechanisms provides a foundation for optimizing battery formation processes, ultimately improving electrochemical performance and durability.^[^ ^46^ ^]^ Zhang et al. identified the ratio of additive mass fraction to electrode surface area as a key descriptor for optimizing the formation process.^[^ ^47^ ^]^ Since electrolyte additives play a crucial role in governing SEI formation mechanisms and growth dynamics, this ratio provides valuable insights for improving battery performance. Based on the same data using additives of fluoroethylene carbonate (FEC) and vinylene carbonate (VC), we further explore the benefits of analyzing and subsequently optimizing battery manufacturing. The model is trained on the formation data of baseline, that is, additive‐free electrolytes. Only the initial charging process (formation) is selected from four formation cycles to maximize the variation of battery performance. The distinct active areas contribute to substantial differences in the estimated diffusion process and SEI growth. Degradation mechanisms that might occur during formation and cycling, such as SEI growth, particle cracking, and dead lithium, are illustrated in **Figure** **5**a. These underlying mechanisms function for the loss of active material (LAM) and inventory lithium (LLI), the two primary degradation modes that shorten battery lifespan.^[^ ^48^, ^49^, ^50^ ^]^

The intrinsic growth of SEI layers and polarization voltage evolution after 50 cycles are isolated by eliminating geometric factors (electrode area and thickness). As presented in Figure 5b,c, higher electrolyte additive concentration per unit anode area (1 mAh cm^−2^) exhibits enhanced SEI formation kinetics, thereby accelerating polarization during cycling. In the analyses of FEC additives with larger electrode volumes, reduced *R_ct_* and increased polarization effects from cycle 1 to cycle 50 (Figure S7, Supporting Information) might indicate the increased electrode area and particle cracking.^[^ ^51^ ^]^ As substantiated in Figure 5d,e, the polarization magnitude demonstrated an additive ratio‐dependent increase under controlled anode geometry, revealing proportional enhancement with VC loading. Under the 3 mAh cm^−2^ anode configuration with 0.1 wt% VC additive, cells exhibited reduced cycling capacity, accompanied by a lower SEI growth and increased polarization. Given the increase of internal resistance during battery aging (Figure S8, Supporting Information), the accumulation of dead lithium is hypothesized to occur under low VC concentrations, while elevated VC loading appears to suppress this parasitic reaction. However, the analysis between the estimated dynamics and the additive ratio is inconclusive because of the lack of experimental validation. Future research directions are recommended to involve designing experiments with tighter control over additive ratios and incorporating more frequent EIS measurements, to track the temporal evolution of SEI resistance with higher resolution and provide stronger validation for the trained model.

Using estimated voltage variables from the first 10 cycles, we forecast aging trends for the subsequent 45 cycles. The voltage values in the lower SOC range (<20%), middle SOC range (20–80%), and upper SOC range (>80%) are intercepted and analyzed via principal component analyses (PCA). Two principal components are adopted as the feature vectors of random forest regression (RFR). The MAPE results are displayed in Figure 5f. Compared to the terminal voltage, the decomposed voltages demonstrate good performance, particularly as *U*_0_ and *U_oc_* achieve lower errors within the middle SOC range. The mapping between the state of health (SOH) and the median values of decomposed voltages within the SOC range of 20–80% is shown in Figure S9 (Supporting Information). Decentralized clusters in the red circles represent a more pronounced polarization reaction. The batteries with a median OCV less than 3.5 V are diagnosed as premature failure. We categorize cells as having failed formation when, alongside an upward shift in all voltage statistics (except OCV), their SOH also declines below 0.8 within the first 10 cycles, thus reaching end‐of‐life prematurely. After discarding prematurely failed batteries, an early prediction is conducted on the remaining cells. As shown in Figure S10 (Supporting Information), the voltage feature associated with internal ohmic resistance outperforms other voltage features in aging predictions, yielding lower prediction errors. The decomposed voltages through PIBMN may benefit battery management and facilitate early failure detection.

### 3.4 Uncertain Aging Prognosis for Diverse Datasets

**Figure 6:**
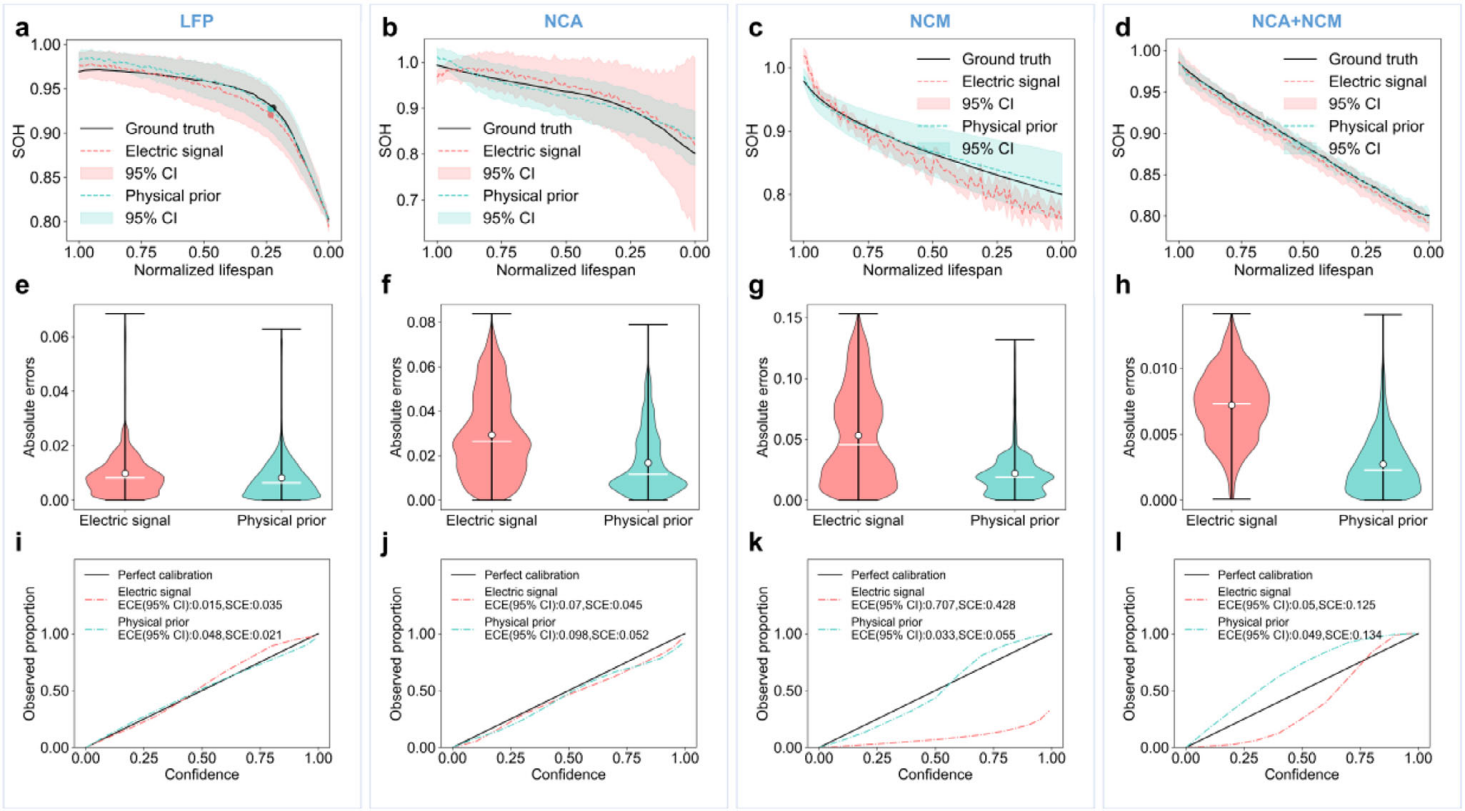
Performance evaluation across cell types. Measured electrical signal and generated physical prior are used as the input features for aging prediction. a–d) Early estimated degradation trajectories with 95% confidence interval (CI). Points on (a) are detected knee points. e–h) Absolute errors on the test set. White point and white line indicate the mean error and the median error, respectively. i–l) Calibration curve and reliability evaluation. The calculated calibration curve above the “perfect calibration” indicates under‐confidence regression; conversely, it indicates over‐confidence regression.

Battery aging prognostics often struggle with generalization across datasets with different degradation trajectories, particularly when encountering novel electrode materials and emerging cell types.^[^ ^52^, ^53^ ^]^ Two publicly available datasets with four cathode materials (detailed in Experimental Section “Dataset Distribution”) undertake the generalizability evaluation of the proposed method, and diverse current rates and ambient temperatures were configured when cycling each cell type. The result of estimated trajectories and evaluated reliability is displayed in **Figure** **6**. After identifying 2RC‐ECM dynamics using dual PIKANs, a deep learning model based on convolutional neural networks and long‐short‐term memory model (CNN‐LSTM) is used to forecast aging trends from electrical signals (current and terminal voltage) and physical priors (*R*_0_, *R*_1_, *R*_2_, *τ*_1_, *τ*_2_). The trajectory uncertainty is quantified via the terminal probabilistic neural network.^[^ ^54^ ^]^ Although the input features are taken from the first 20 cycles, the resulting aging trend is projected over dozens to thousands of cycles. In order to unify the training set, the cycle numbers are normalized to a fractional lifespan (from 1 to 0), and the SOH values are resampled at 100 equally spaced points along this normalized axis. Normalized lifespan in Figure 6a–d is defined as 1 – (current cycles / total cycles). Total cycles mean the cycle number when SOH approaches 0.8. More information for implementing PIKAN and this uncertain predictive model is detailed in Experimental Section “Training Configuration” and Note S5 (Supporting Information).

Compared to the electrical signal‐based feature baseline, physical prior‐based aging characteristics perform more reliably and accurately in early aging prediction. When assessing the method using LFP batteries of the MIT‐Stanford dataset, interest arises in the degradation mechanism responsible for the capacity drop before SOH approaches 0.8, which leads to a knee point in the capacity decay curve. Knee points identified by the Bacon–Watts model^[^ ^55^ ^]^ on the predicted and real aging trajectories are marked in Figure 6a, and these points nearly coincide. From the validation results of four cell types, the predicted aging curve based on physical prior knowledge aligns more closely with the real aging trend in Figure 6a–d, and the statistical error is smaller in Figure 6e–h, which indicates the effective improvement in battery management when considering uncovered mechanisms. Moreover, the calibration curves in Figure 6i–l prove the reliability and trustworthiness of aging prediction based on PIBMN‐solved physical dynamics. Expected calibration error (ECE) in the 95% CIs and statistical calibration error (SCE) using two aging features are also compared. Despite slightly larger calibration errors in physical prior‐based estimation compared to electrical signal‐based estimation, especially for LFP and NCA cells, the markedly reduced error on NCM cells underscores the generalizability of our proposed approach for aging prediction.

### 3.5 Discussion

In summary, this study reformulates process kinetics for rechargeable batteries into fast‐slow time‐constrained PIBMN. This allows for a rapid reparameterization of cells with unknown chemistry without the need to open cells. The generalized model is transferable across cell geometries, chemistries, and protocols, allowing for transient dynamics after as little as a formation cycle and even allowing for a smooth translation from a GITT to a drive cycle. Leveraging the physical constraints of the built ARX model, PIBMN is given more explainability than the conventional intelligent approaches and potentially even offers input for high‐fidelity P2D and P4D models. The estimated SEI resistance and polarization voltage offer a novel approach to battery characterization, accelerating the design of electrodes and electrolytes. Real‐time identification of OCV and ohmic resistance enhances manufacturing quality control and enables early aging prediction.

Compared to other recent physics‐informed approaches, as summarized in **Table** **1**, PIBMN is essentially based on ECM models at different orders. The ability to adaptively estimate the time constant in real‐time allows the PIBMN to play a role like EIS testing in different scenarios, thus expediting mechanism diagnosis and lifetime prediction. Different from other PIML approaches for SOH prediction^[^ ^27^ ^]^ and degradation diagnostics,^[^ ^56^ ^]^ the input for model implementation is the raw electrical signal, bypassing additional feature engineering. Compared to PIML using SPM or P2D as the surrogate model,^[^ ^57^, ^58^ ^]^ PIBMN enables an enhanced understanding of actual performance by providing instantaneous resistance components, even without directly solving for the concentration distribution based on electrode design parameters. Overall, PIBMN is an easily applicable, trustworthy, and accurate battery simulator that can be embedded in most practical scenarios.

**Table 1:** Comparison with recent physics‐informed battery modeling approaches.

Method	Surrogate model	Neural network	Input variables	Output variables	Application
PINN4SOH^[^ ^27^ ^]^	SOH decay rates	MLP	SOC, temperature, health indicators, and other factors	SOH	SOH prediction
Navidi et al.^[^ ^56^ ^]^	Half‐cell model	MLP	dQ/dV	Capacity, LLI	Degradation diagnostic
PI‐DeepONets^[^ ^57^ ^]^	SPM	Neural operator	Current, Diffusivities	Li‐ion concentration Voltage response	Design of experiments
PE‐FNO^[^ ^58^ ^]^	SPM	Fourier neural operator	Current, SOC, Radius, Diffusivities	Li‐ion concentration Voltage response	Battery simulation
PIBMN (This study)	ECM	MLP, KAN	Current, Voltage (historical)	Voltage response, resistances, capacitances, time constants	Real‐time dynamic monitoring of OCV, aging states, and failure modes

## 4 Experimental Section

### Dataset Description

Battery datasets for method validation are listed in **Table** **2**. The cell types used in Section 3.1 are LG M50 and Maxell ML2016, with datasets generated by PyBaMM simulations and lab‐scale GITT tests, respectively. The voltage response of LG M50 cells under US06 driving protocols is generated (see Note S6, Supporting Information). The GITT protocol simulated in PyBaMM applies a C/10 or C/20 constant‐current charge or discharge for 30 minutes, followed by a 1 h open‐circuit relaxation period (see Note S6, Supporting Information). The US06 driving cycle and GITT experiments on Maxell ML2016 are performed using the same procedure under different ambient temperatures. The batteries in Sections 3.2 and 3.3 are CR2032 coin cells with a lithium nickel oxide (LNO) positive electrode and a graphite negative electrode. They are manufactured using the automatic battery assembly system (AUTOBASS).^[^ ^47^, ^59^ ^]^ Additional details, including manufacturing specifications, formation protocols, and cycling tests, are provided in our previous work.^[^ ^42^ ^]^ Two cycling datasets in Section 3.4 are published by MIT‐Stanford University^[^ ^16^ ^]^ and the KIT‐Tongji University.^[^ ^60^ ^]^ The MIT‐Stanford dataset includes 124 LFP 18650 batteries with different fast charging conditions and 3C discharging current rates. The KIT‐Tongji dataset includes three kinds of commercial batteries that were cycled at different temperatures. For all battery datasets in this study, measured current and voltage signals are resampled to a temporal resolution of 1 s using linear interpolation. The ratio of training to testing batteries is usually set at 8:2.

**Table 2:** Battery datasets used in this study.

Cell type	Positive/Negative materials	Number	Temperature	Scenario	Source
LG M50	NMC/C‐SiOx	3	25 °C	GITT test; Driving	PyBaMM simulation
Maxell ML2016	MnO/Li‐Al	31	10, 15, 20 °C, Room temperature	GITT test; Driving	Lab test
CR2032	LNO/Graphite	103	Room temperature	Formation, Cycling test	[47]
18650	LFP/Graphite	124	30 °C	Fast charging	[16]
LG INR 35E	NCA/C‐Si	65	25, 35, 45 °C	Cycling test	[60]
LG INR MJ1	NCM/C‐Si	55	25, 35, 45 °C	Cycling test	[60]
Samsung INR 25R	NCM+NCA/Graphite	9	25 °C	Cycling test	[60]

### Loss Function

As depicted in Figure 2, *U^t^* represents the terminal voltage, which can be directly measured. *U^SD^* is the slow‐dynamic component voltage composed of OCV and diffusion potential. *U^FD^* is the component in the fast‐dynamic component voltage that includes ohmic resistance voltage and charge transfer voltage. The FD Net estimates the present terminal voltage based on the previous terminal voltage, current, and estimated slow‐dynamic voltage information. The SD Net predicts *U^SD^*. The automatic difference for each mode input is embedded in the governing equations to generate physics loss. The total loss for backpropagation and optimization on each network is calculated by:

(1)

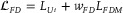



(2)

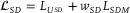

where $L_{FD}$ and $L_{SD}$ are the loss functions of fast‐dynamic and slow‐dynamic neural networks. *w_FD_* and *w_SD_* indicate the weight of physics loss, which automatically increases as the iteration epoch increases: *w* = exp(1‐(Current epoch number)/(Total epoch number)). The data loss 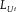 and 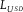 are calculated based on mean squared errors (MSE). The constraints to calculate physics loss *L_FDM_* are shown in Equation S3 (Supporting Information). The SD constraints for the standard 2RC ECM to calculate physics loss *L_SDM_* are described in Equation S6 (Supporting Information). When the surrogate model is the higher‐order ECM with Warburg element, the SD constraints to compute physics loss *L_SDM_* are described in Equation S9 (Supporting Information). The MSE of the estimated OCV and time constant can also be added to the physics loss *L_SDM_* for hard control.

### Training Configuration—Configuration for PIBMN on Different Datasets

To ensure real‐time application of the PIBMN model in an online battery monitoring system, the model is typically pretrained in advance. During deployment, it can either be fine‐tuned if further adaptation is required, or the physical parameters can be directly estimated without backpropagation. When fine‐tuning is performed, the AdamW optimizer is used with a learning rate (LR) of 1×10^−6^. The pretraining configurations for different datasets are provided in **Table** **3**. The initial LR is 1e‐3, and the weight decay is 1e‐4. AdamW and Radam optimizers are implemented using the Torch optimizer library. It should be noted that the network nodes and hyperparameters described are intended solely to facilitate the reproduction of the results in this study. The algorithms for model selection or hyperparameter optimization can be used to identify the optimal model configuration.

**Table 3:** Pretraining configuration on different datasets.

Cell type	FD Net [KAN]	SD Net [KAN]	FD Net [MLP]	SD Net [MLP]	Optimizer	Batch size	Total epoch
LG M50	(m,1)	(n,1)	(m,2,1)	(n,2,1)	AdamW	512	50
Maxell ML2016	(m,4,2,1)	(n,2,2,1)	(m,2,2,1)	(n,2,2,1)	Radam+Lookahead	512	10
CR2032	(m,4,2,1)	(n,2,2,1)	–	–	AdamW	512	50
LFP 18650	(m,1)	(n,1)	–	–	Radam+Lookahead	256	100
LG INR 35E	(m,1)	(n,1)	–	–	Radam+Lookahead	256	100

### Training Configuration—Configuration for Early Aging Prediction

The CNN‐LSTM uncertain learning architecture used for early aging prediction is illustrated in Note S5 (Supporting Information). The model is optimized using Adam with LR = 10^−3^. The total epoch number is 100. Batch sizes are set to 64 for LFP, 16 for NCA, 16 for NCM, and 4 for NCM+NCA. The input dimensions are 2 for electrical signal inputs and 5 for physical prior inputs. To accelerate computation, all electrical signals are resampled to a 30‐s interval. Our experimental results indicate that the predictive performance varies with different SOC ranges of electrical signals and physical prior inputs. Automated selection of the optimal input range represents a valuable direction for future research.

### MLP and KAN

Conventional MLP is usually modeled via fixed activation functions on nodes and learnable weights on edges, which can be formulated as:

(3) $$\mathrm{MLP}\left(x\right)=\left(W_{L}\circ \sigma_{L-1}\circ \cdots \circ W_{2}\circ \sigma_{1}\circ W_{1}\right)\left(x\right)$$

where **W** *_i_* and *σ_i_* are the weight matrix and activation function at the *i* ^th^ MLP layer. In this study, MLP is performed via linear function and activation functions in the Torch environment.

Basic KAN^[^ ^61^ ^]^ is modeled via learnable activation functions on edges and the sum operation on nodes, which can be formulated as:

(4) $$\mathrm{KAN}\left(x\right)=\left(\Phi_{L}\circ \Phi_{L-1}\circ \cdots \circ \Phi_{2}\circ \Phi_{1}\right)\left(x\right)$$

where **Φ** *_i_* is the function matrix corresponding to the *i* ^th^ KAN layer and consists of parameterized residual activation functions, each implemented as the sum of a silu activation function and a learnable B‐spline function. The mathematical expression of the residual activation function is defined as:

(5) $$\phi =w_{s}\cdot spline\left(x\right)+w_{b}\cdot silu\left(x\right)$$

where *w_s_* and *w_b_* are the weights of the B‐spline function and the silu activation function.

KAN linear is regarded as the convolutional KAN kernel in the recent research^[^ ^38^ ^]^ and offers an improvement in parameter efficiency and expressive power. The related software can help to implement KAN conveniently, like MLP in the Torch environment (https://github.com/AntonioTepsich/Convolutional‐KANs).

## Conflict of Interest

The authors declare no conflict of interest.

## Supporting information



Supporting Information

## Data Availability

Codes for the PIBMN framework are open‐sourced on GitHub: https://github.com/SLJME42/PIBMN. The battery formation and cycling data are available at https://zenodo.org/records/11060629. Requests for further information and resources should be directed to and will be fulfilled by the lead contact, Helge S. Stein (helge.stein@tum.de).
