# Supplementary material for: Continuous Physics‐Informed Learning Expedited Battery Mechanism Decoupling
Source: Adv Sci (Weinh). 2025 Oct 27;13(1):e06772. doi: 10.1002/advs.202506772 (PMC12767122; doi:10.1002/advs.202506772)

# **Supporting Information**

**Continuous Physics-informed Learning Expedited Battery Mechanism Decoupling**

*Shanling Ji*, Jun Yuan, Bojing Zhang, Aleksei Sanin, Leon Merker, Zhisheng Zhang, Jianxiong Zhu*, and Helge Sören Stein**


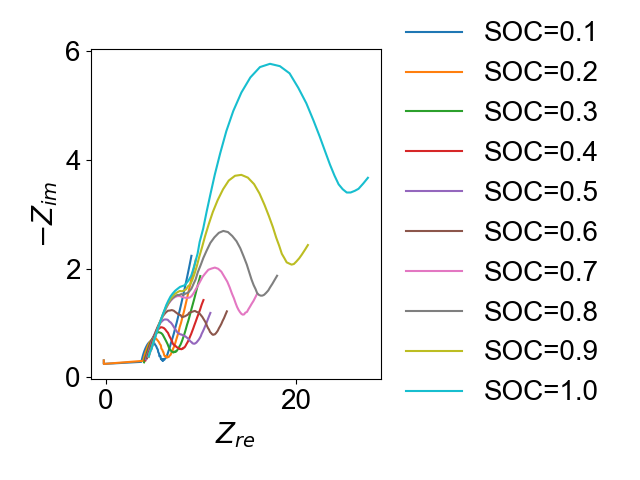

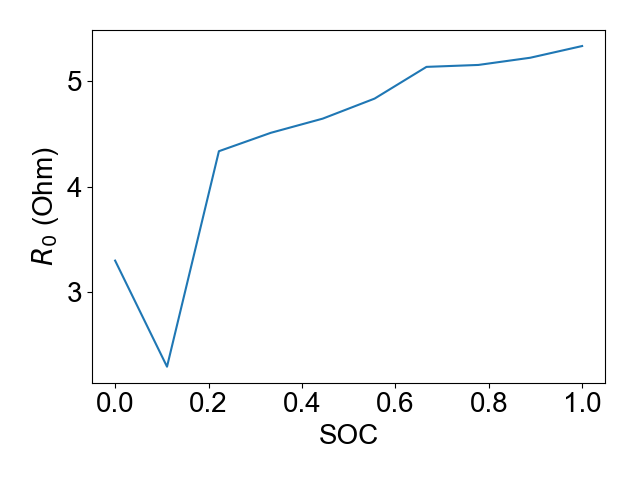


**Figure S1.** EIS tests on the Maxell ML2016 coin cell.


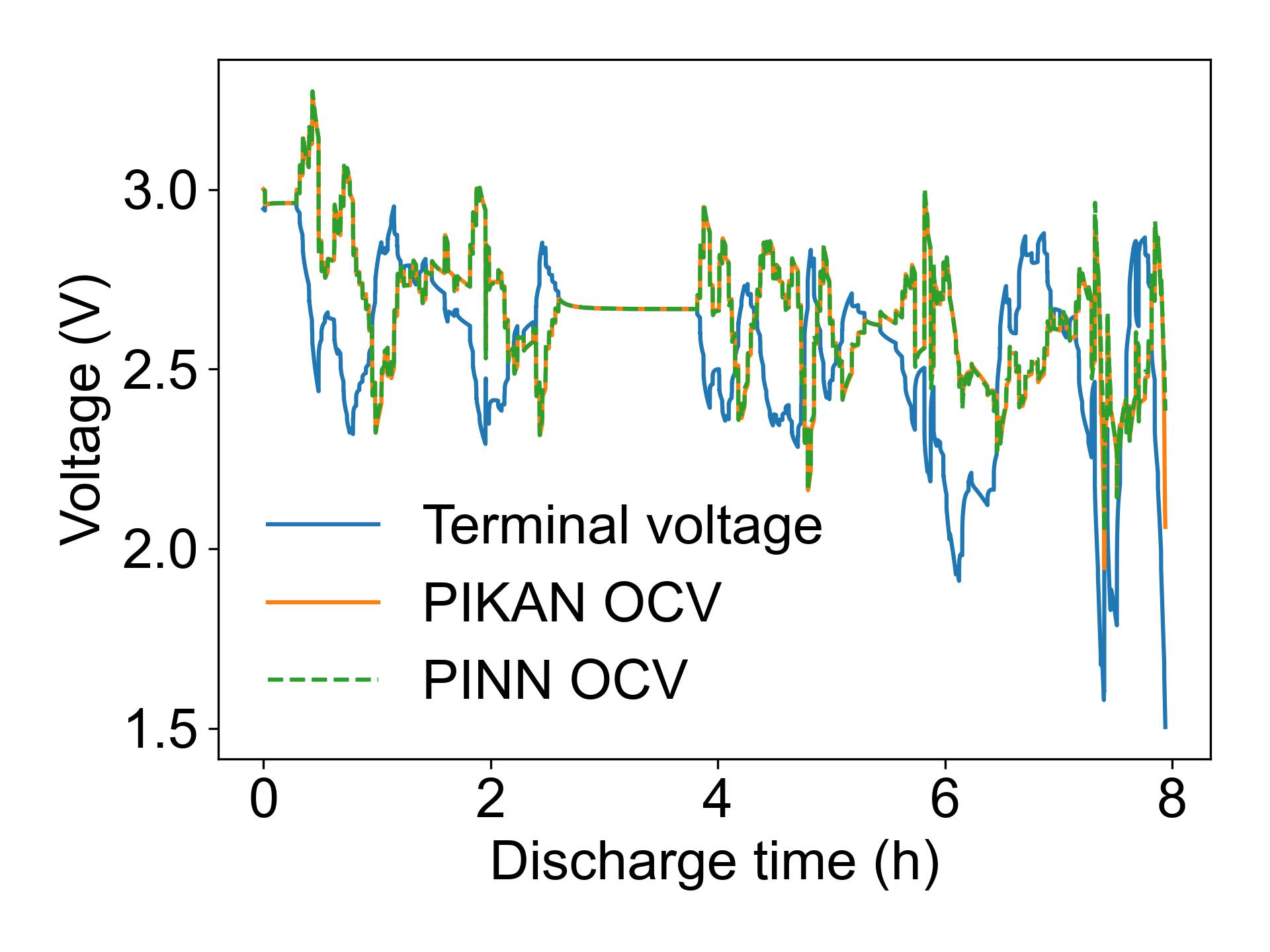


**Figure S2.** OCV estimation under driving conditions of Maxell ML2016 coin cell.


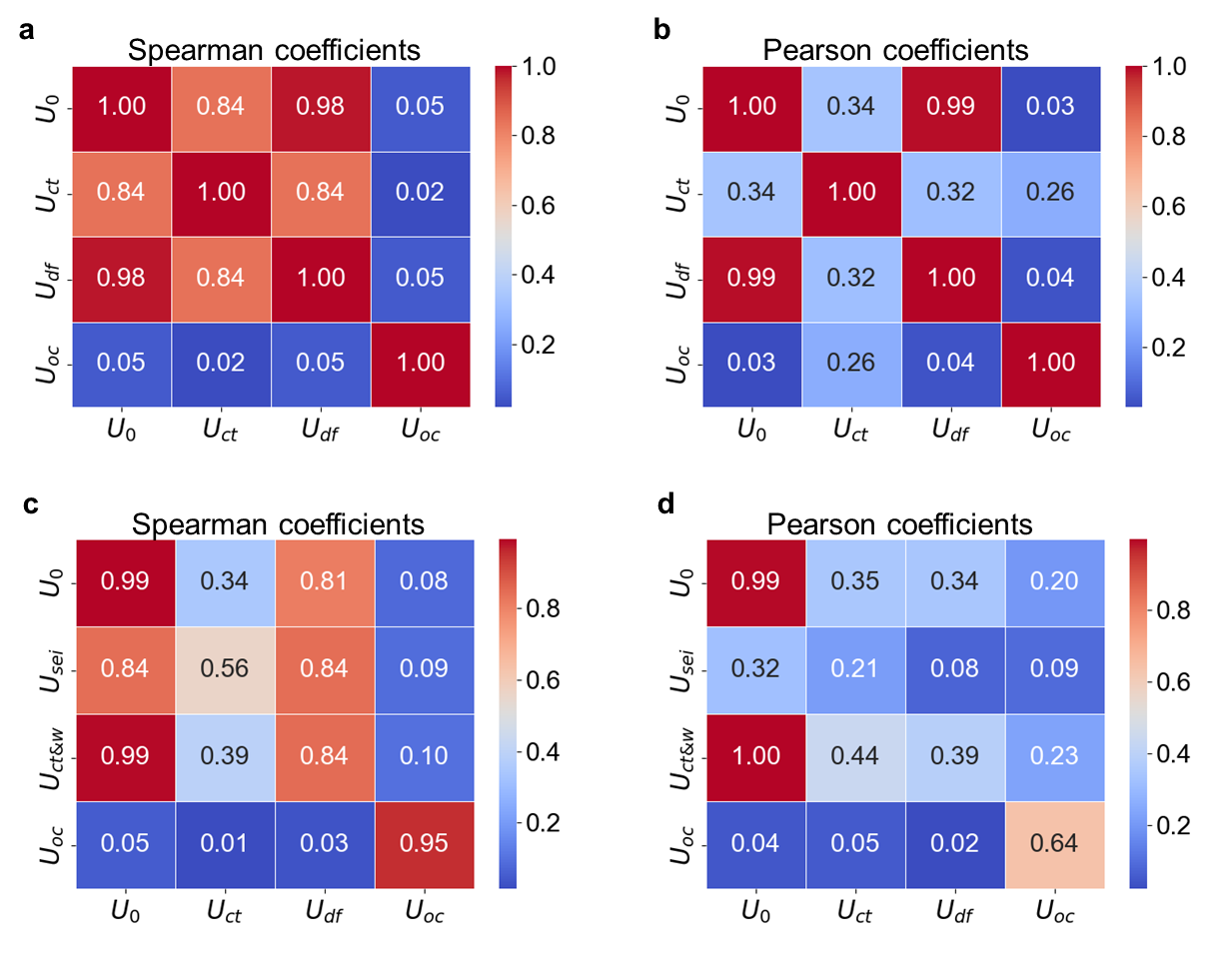


**Figure S3.** Correlation analysis among voltage components. (a) and (b) are for the same 2RC ECM. (c) and (d) are for different ECM.


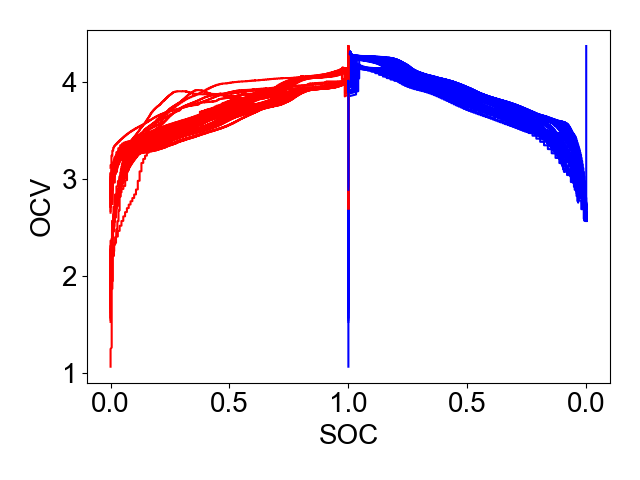

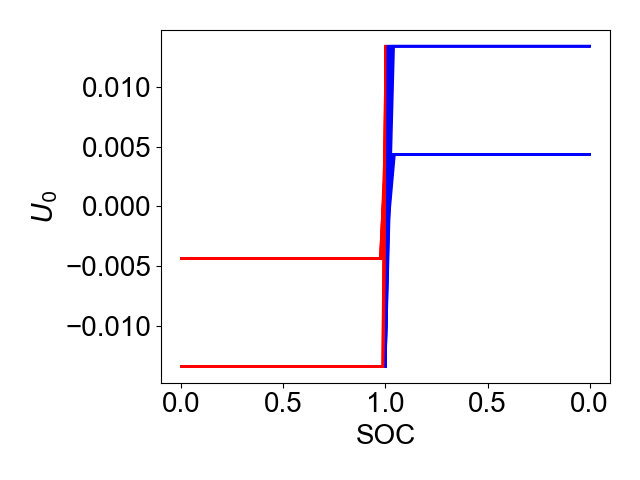


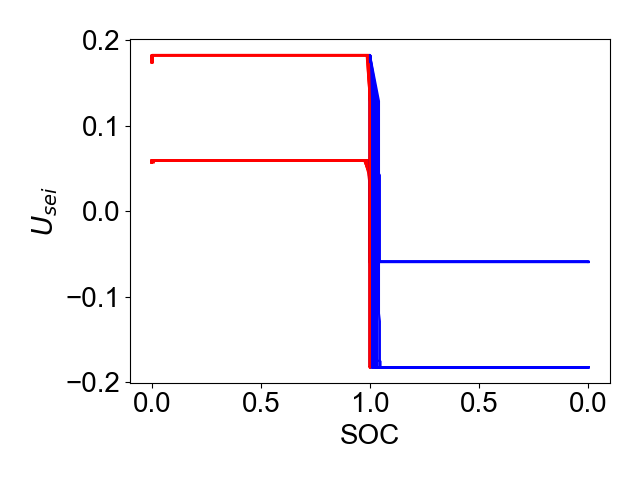

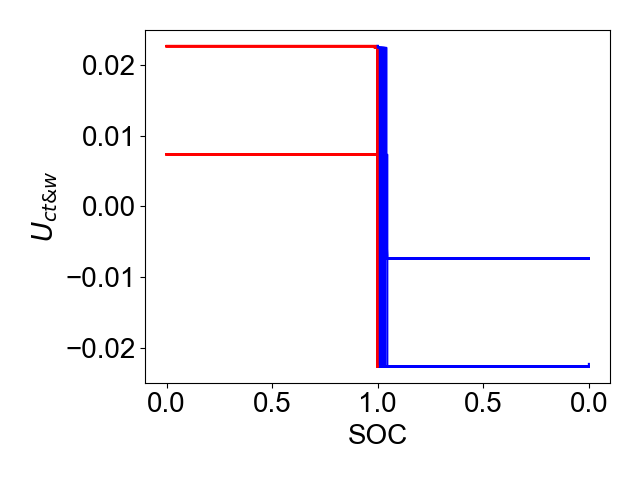


**Figure S4.** PINN performance on extended ARX model. Red line is charge. Blue line is discharge. The database of baseline electrolyte also includes two anode areas. Ohmic overpotential is not accurately estimated since it should be positive value during charging.


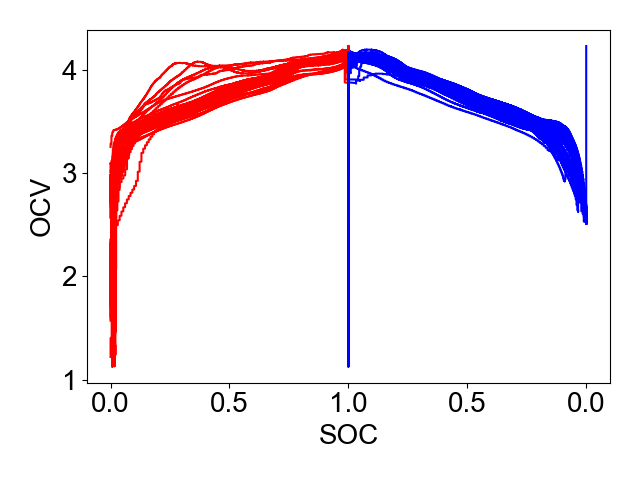

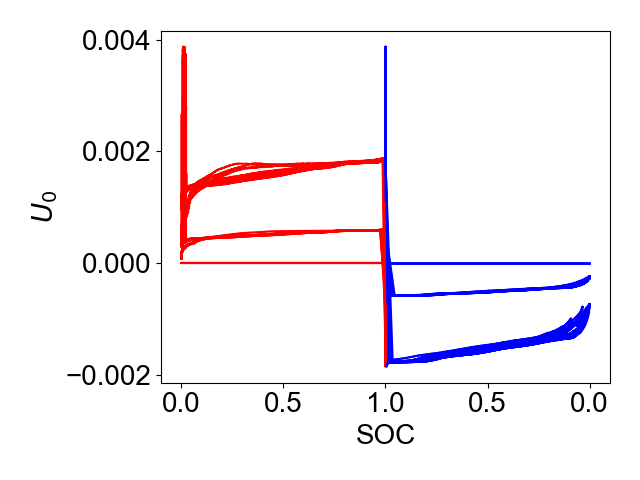


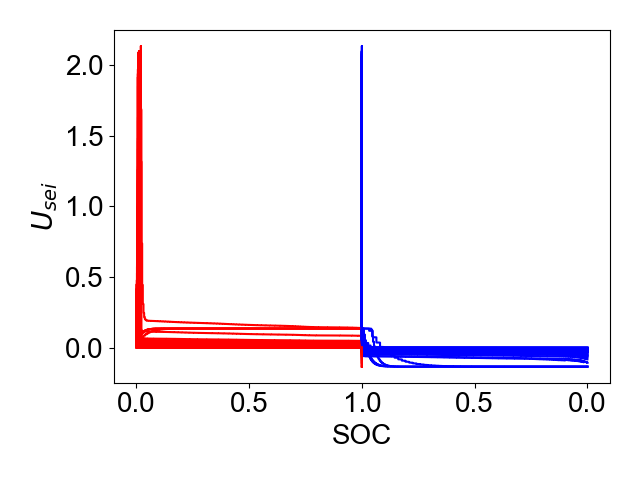

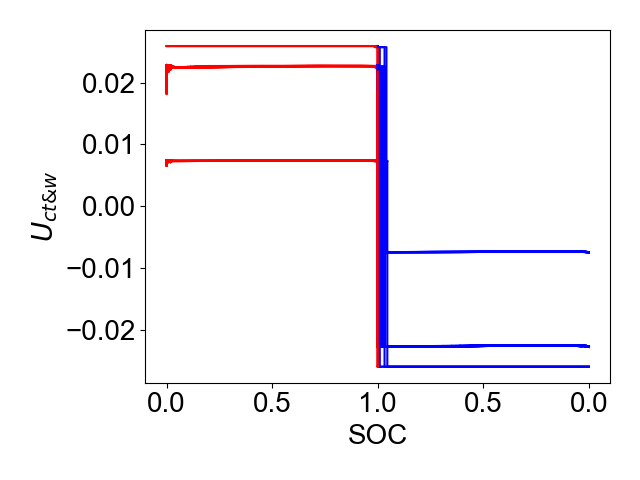


**Figure S5.** PIKAN performance on extended ARX model. Red line is charge. Blue line is discharge. The database of baseline electrolyte also includes two anode areas.


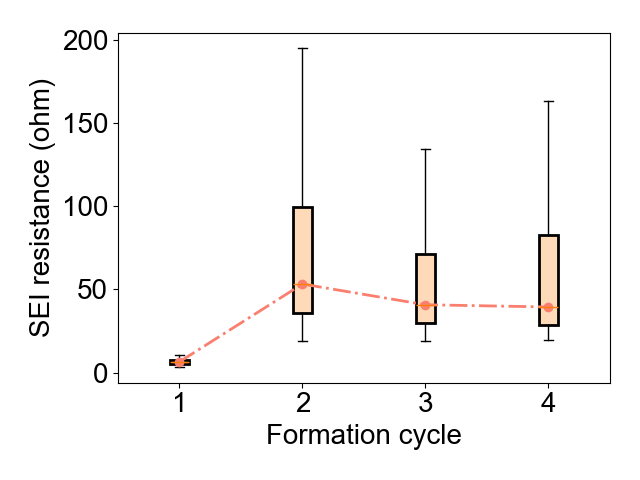


**Figure S6.** SEI resistance growth during formation.


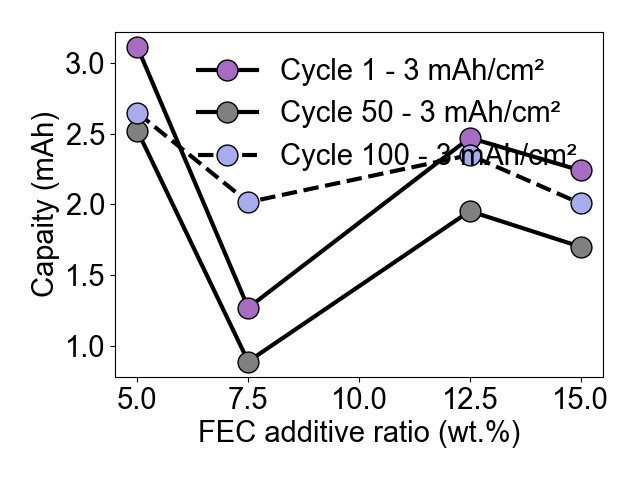

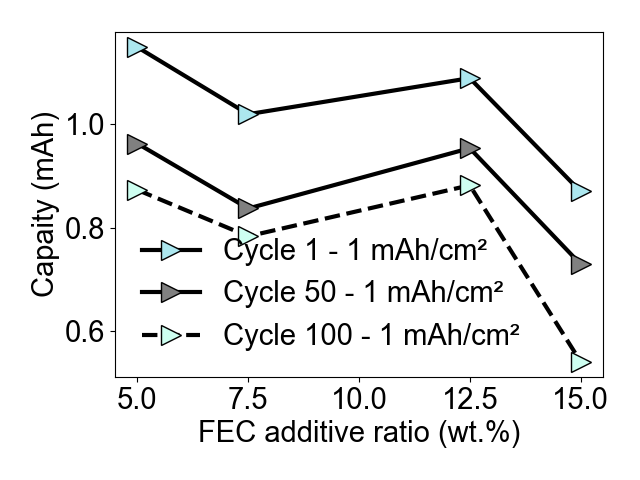

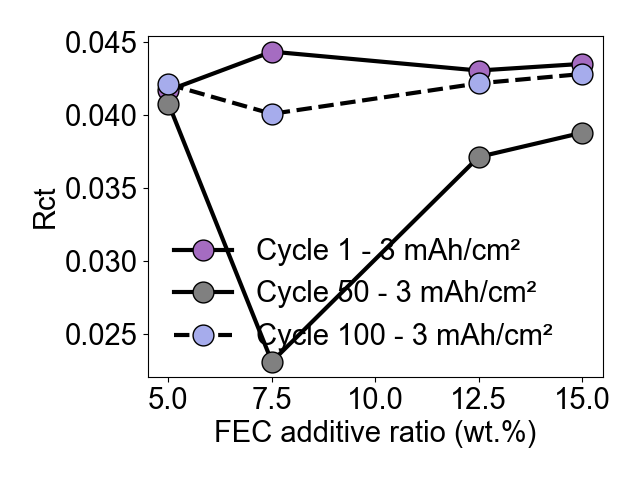

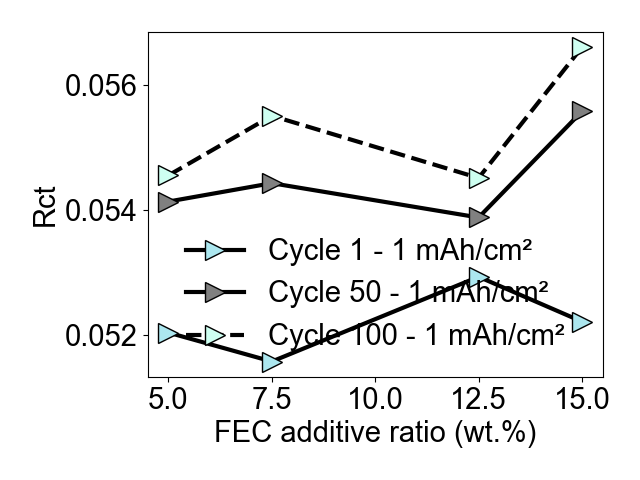


**Figure S7**. Capacity and *R_ct_* at different FEC additive ratios and electrode volumes.


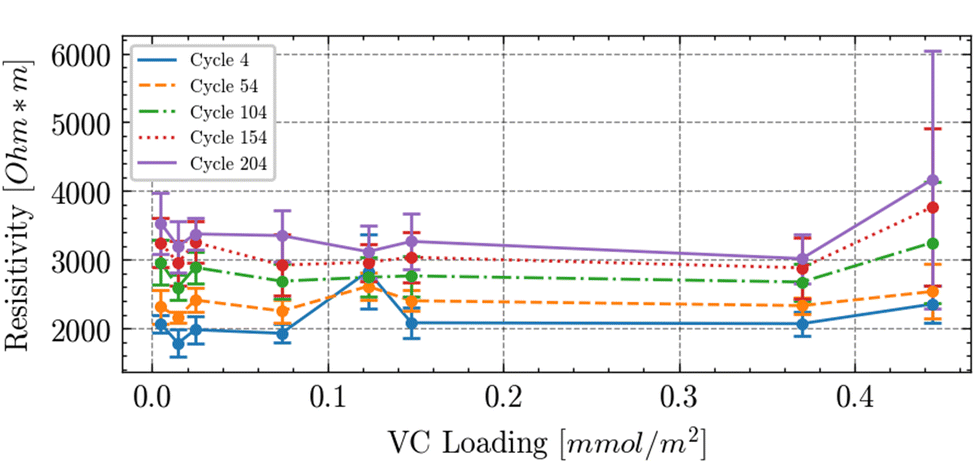


**Figure S8**. Resistivity variation on different VC loadings.^[47]^


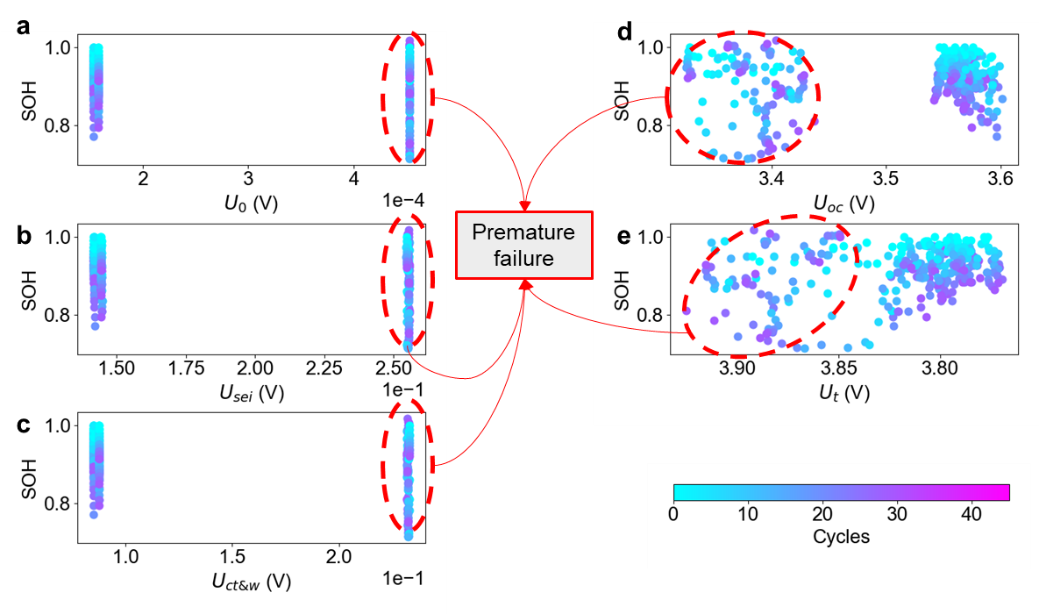


**Figure S9**. Battery premature failure diagnosis through decomposed voltages.


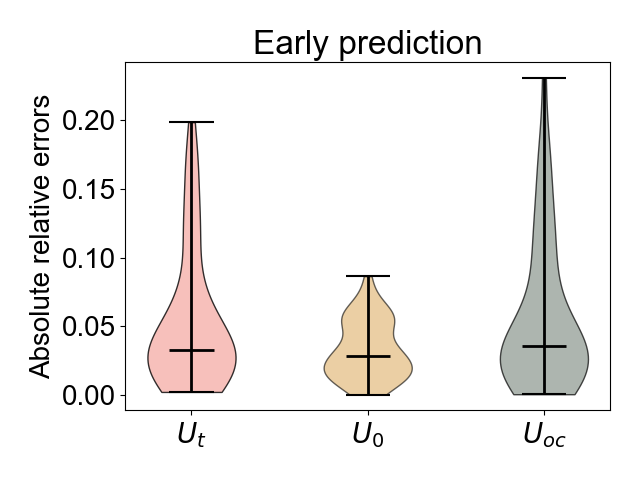


**Figure S10**. Battery premature failure diagnosis through decomposed voltages.

**Note S1: Battery ARX model informed networks**

The ARX model of second-order RC ECM can be derived as follows^[36]^:

$$u_{t}\left(k\right)=c_{1}u_{t}\left(k-1\right)+c_{2}i_{t}\left(k\right)+c_{3}i_{t}\left(k-1\right)+u_{SD}\left(k\right)-c_{1}u_{SD}\left(k-1\right)$$

With this ARX model, the input-output pair $\left(\mathbf{x}_{FD},u_{t}\right)$ for the FD Net is determined and input vector $\mathbf{x}_{FD}$ involves five values of last moment and this moment. Terminal voltage $u_{t}$ at this moment is estimated. The physical representation of automatic differentiation is:

$$\left\{\begin{matrix} \frac{\partial u_{t}}{\partial x_{FD,1}}=c_{1}=\frac{\tau_{1}}{T_{s}+\tau_{1}} \\ \frac{\partial u_{t}}{\partial x_{FD,2}}=c_{2}=R_{0}+\frac{T_{s}R_{1}}{T_{s}+\tau_{1}} \\ \frac{\partial u_{t}}{\partial x_{FD,3}}=c_{3}=\frac{-R_{0}\tau_{1}}{T_{s}+\tau_{1}} \end{matrix}\right.$$

Physical loss constraints are acquired from automatic differentiation:

$$\left\{\begin{matrix} \frac{\partial u_{t}}{\partial x_{FD,4}}=1 \\ \frac{\partial u_{t}}{\partial x_{FD,1}}+\frac{\partial u_{t}}{\partial x_{FD,5}}=0 \\ R_{0}\frac{\partial u_{t}}{\partial x_{FD,1}}+\frac{\partial u_{t}}{\partial x_{FD,3}}=0 \\ \begin{matrix} \frac{\partial u_{t}}{\partial x_{FD,1}},\frac{\partial u_{t}}{\partial x_{FD,2}}>0 \\ \frac{\partial u_{t}}{\partial x_{FD,3}},\frac{\partial u_{t}}{\partial x_{FD,5}}<0 \end{matrix} \end{matrix}\right.$$

wherein $R_{0}$ needs to be initialized. When considering strong constraints, $c_{2}$ value can also be initialized.

Likewise, we derived ARX model for slow dynamics as follows:

$$u_{SD}(k)=b_{1}u_{SD}(k-1)+b_{2}i_{t}(k)+u_{oc}(k)-b_{1}u_{oc}(k-1)$$

where physical representation of automatic differentiation is:

$$\left\{\begin{matrix} \frac{\partial u_{SD}}{\partial x_{SD,1}}=b_{1}=\frac{\tau_{2}}{T_{s}+\tau_{2}} \\ \frac{\partial u_{SD}}{\partial x_{SD,2}}=b_{2}=\frac{\tau_{2}R_{2}}{T_{s}+\tau_{2}} \end{matrix}\right.$$

Hence, the input-out pair for the SD Net is $\left(\mathbf{x}_{SD},u_{SD}\right)$ and $\mathbf{x}_{SD}$ involves four values of last moment and this moment. Slow-dynamic voltage $u_{SD}$ at the latest moment is estimated. Physical loss constraints for slow-dynamic networks are acquired from automatic differentiation:

$$\left\{\begin{matrix} \frac{\partial u_{SD}}{\partial x_{SD,3}}=1 \\ \frac{\partial u_{SD}}{\partial x_{SD,1}}+\frac{\partial u_{SD}}{\partial x_{SD,4}}=0 \\ \frac{\partial u_{SD}}{\partial x_{SD,1}},\frac{\partial u_{SD}}{\partial x_{SD,2}}>0 \end{matrix}\right.$$

When the model is updated with newly sampled current and terminal voltage, kinetic parameters can be estimated with automatic differentiation based on Equation S2 and S5. In the second-order RC model, two RC branches represent charge transfer and diffusion processes, respectively. The time constants $\tau_{1}$ and $\tau_{2}$ can be constrained to distinguish fast-slow time scales for the temporal dual networks. Admittedly, the approach performance is not devoid of imperfections when considering parameter initialization, although the parameter amount that requires initialization is declined to two ($R_{0}$ and $c_{2}$) in the FD Net. Bayesian optimization is also adopted to assist in initializing^[20]^.

**Note S2: FAIR comparison between PINN and PIKAN**

1. *Training convergence*

The convergence rates of PINN and PIKAN with identical layer depths are compared in Figure S11 using data from the MAXELL 2016 batteries. Under equivalent architectures, PIKAN exhibits a higher computational cost. PINN achieves an MAPE of 0.01% within 10 epochs, whereas PIKAN requires 15 epochs to reach the same accuracy.

~~
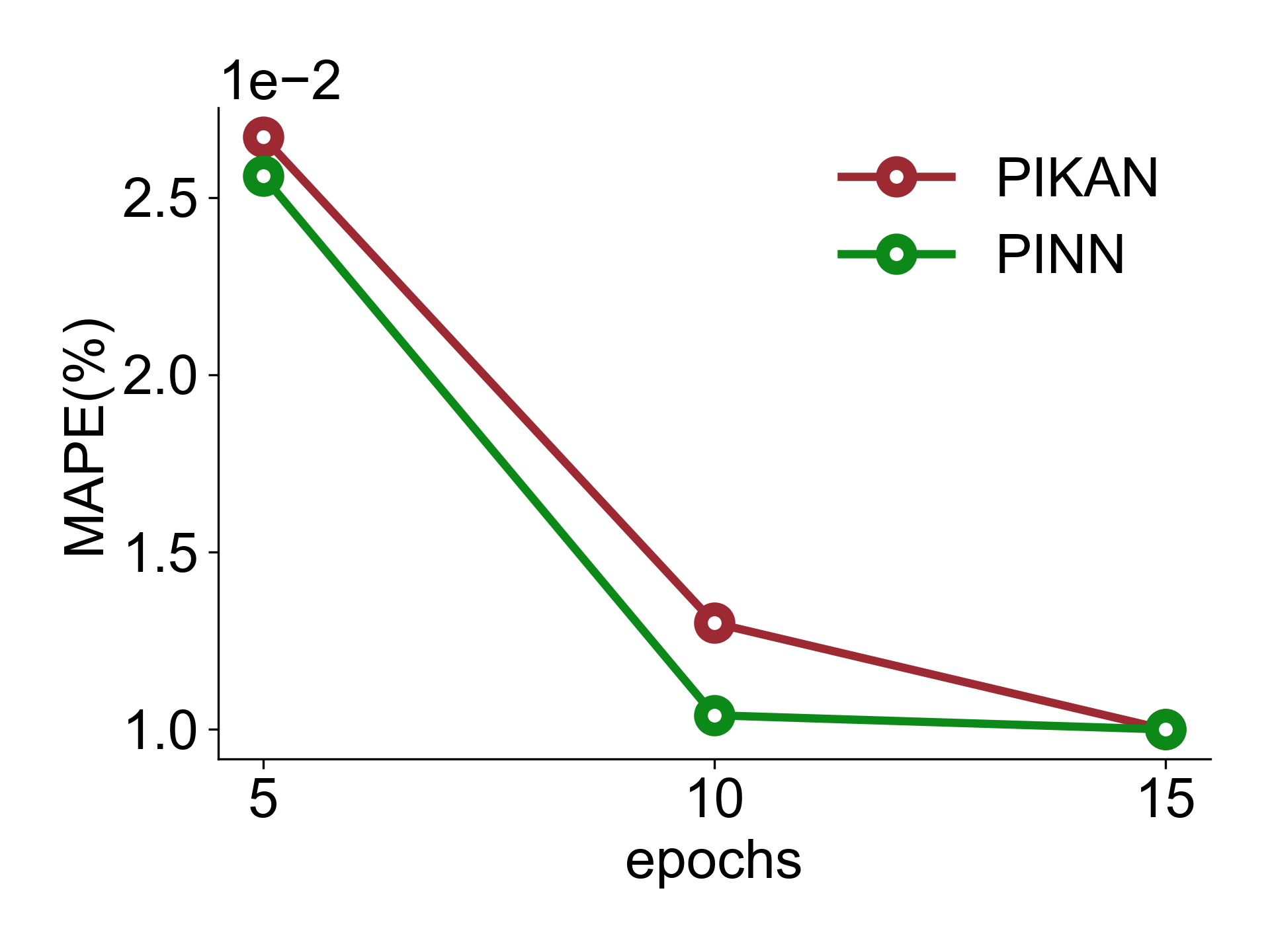
~~

**Figure S11**. Comparison of PINN and PIKAN in convergence speed.

1. *Stability*

Actual driving cycles often induce significant fluctuations in electrical signals, which poses a challenge to the stable implementation of both PINN and PIKAN. While increasing the network depth uniformly leads to convergence issues in PIKAN, these can be mitigated by reducing the number of layers and employing robust optimizers. This configuration not only resolves the convergence problem but also enables PIKAN to achieve higher accuracy, as evidenced in Figure 3c.

1. *Performance with different activation functions*

**Table S1**. PINN with different activation functions.

| Activation function | Convergence | Nonlinearity | MAPE |
| --- | --- | --- | --- |
| Baseline | Yes | No | 0.0225 |
| +ReLU | No | - | Inf |
| +Sigmoid | Yes | Yes | 0.0225 |
| +Tanh | Yes | Yes | 0.0225 |


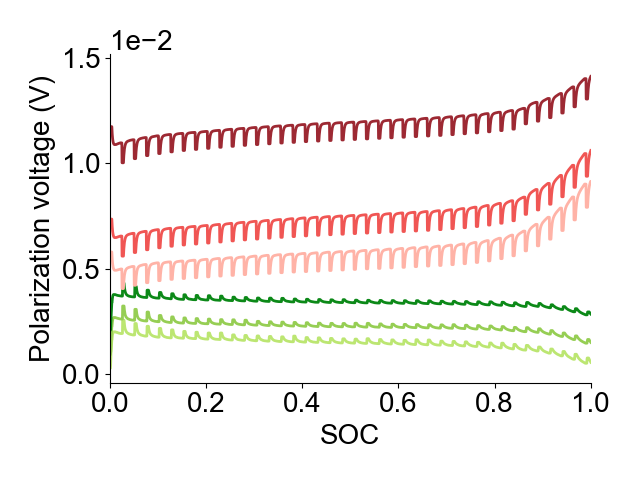


**Figure S12**. PINN (Green) implementation with Sigmoid activation.


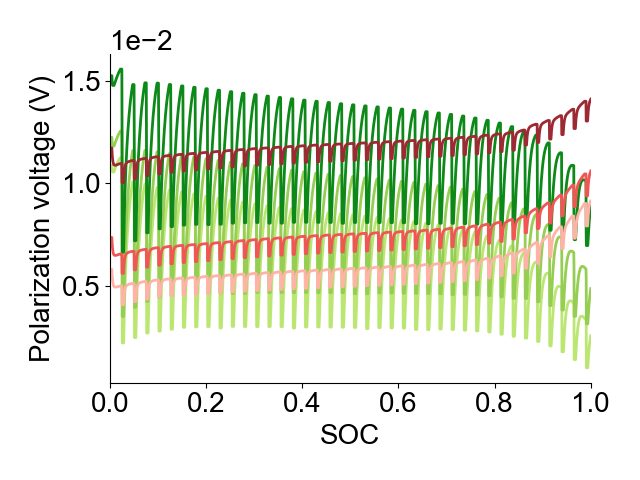


**Figure S13**. PINN (Green) implementation with Tanh activation.

**Note S3: ARX model considering SEI and Warburg element**

The ECM considering Warburg element can simultaneously simulate SEI layer, charge transfer and diffusion processes. The ohmic resistance and RC branch with SEI resistance and capacitance are divided into fast-dynamic networks, while the RC branch including charge transfer processes and Warburg element is divided into slow-dynamic networks. Therefore, the ARX model for the fast dynamic-network is the same as Equation S1, but the physical meanings of *R*_1_ and *C*_1_ are changed into the SEI resistance and capacitance. The ARX model with Warburg element is:

$$u_{SD}(k)=a_{1}u_{SD}(k-1)+u_{oc}(k)-a_{1}u_{oc}(k-1)+a_{2}i_{t}(k)+a_{3}i_{t}(k-1)$$

where the physical meaning of automatic differentiation is:

$$\left\{\begin{matrix} \frac{\partial u_{SD}}{\partial x_{SD,1}}=a_{1}=\frac{0.5+C_{2}\sigma_{w}+0.5C_{2}R_{2}}{1+C_{2}\sigma_{w}} \\ \frac{\partial u_{SD}}{\partial x_{SD,3}}=a_{2}=\frac{\sigma_{w}}{1+C_{2}\sigma_{w}} \\ \frac{\partial u_{SD}}{\partial x_{SD,4}}=a_{3}=-\frac{0.5R_{2}}{1+C_{2}\sigma_{w}} \end{matrix}\right.$$

Physical loss functions for slow-dynamic networks are calculated by following constraints:

$$\left\{\begin{matrix} \frac{\partial u_{SD}}{\partial x_{SD,2}}=1 \\ \frac{\partial u_{SD}}{\partial x_{SD,1}}+\frac{\partial u_{SD}}{\partial x_{SD,3}}=0 \\ \frac{\partial u_{SD}}{\partial x_{SD,1}},\frac{\partial u_{SD}}{\partial x_{SD,4}}>0 \\ \frac{\partial u_{SD}}{\partial x_{SD,3}},\frac{\partial u_{SD}}{\partial x_{SD,5}}<0 \end{matrix}\right.$$

**Note S4: ICA for SEI formation analysis using decomposed voltages**
Here, we analyze the formation process for ensembled coin cells. The 10^th^ cell with baseline electrolyte is adopted to conduct the voltage decomposition and IC, as displayed in Figure S15. During high voltage (Ut>3.8V) or higher SOC range, resistance to Li-ion transport and insertion into negative electrode materials increases, which may be the reason that dQ/dU_sei_ decreases into negative values.


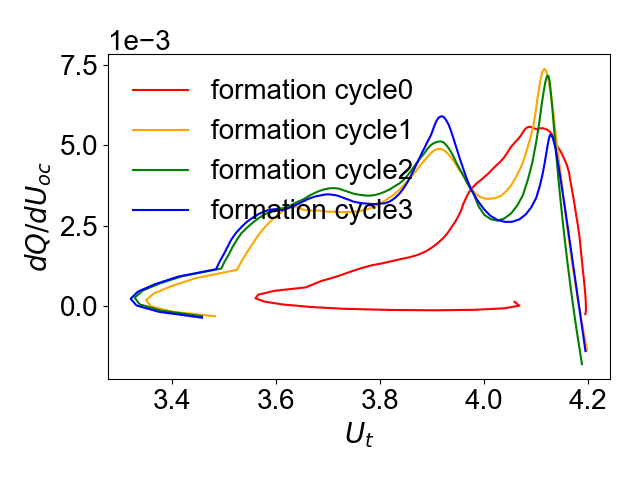

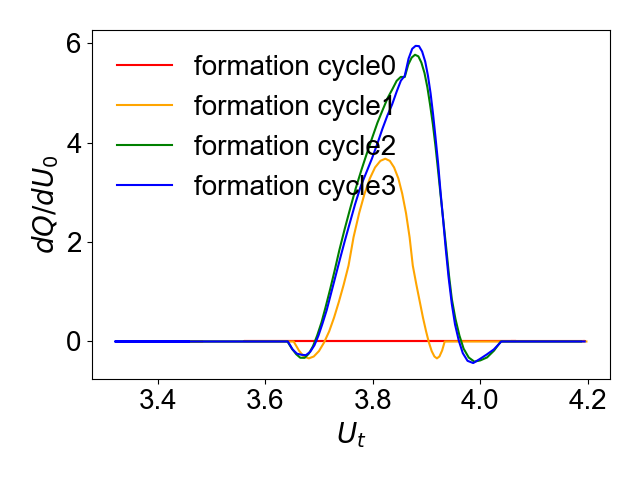


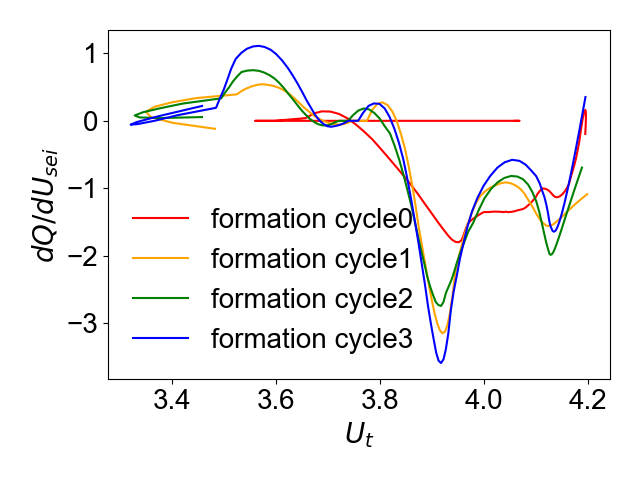

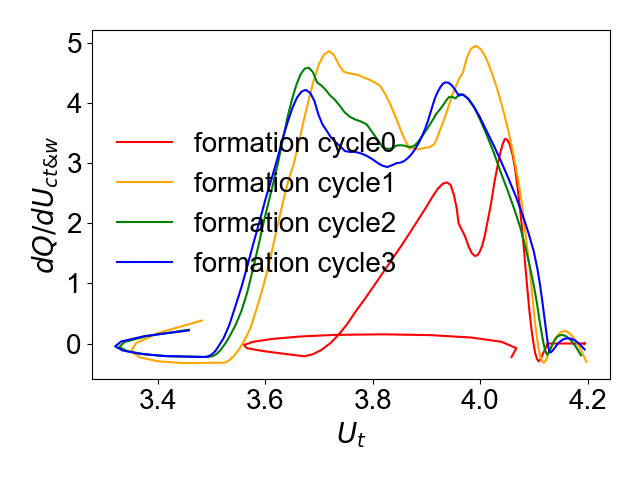


**Figure S14**. ICA with decomposed voltages.

**Note S5: Uncertain learning architecture for aging prediction**

For NCA and NCM cells.

| **Module** | **Layer** | **Input shape** | **Output shape** | **Kernel size** |
| --- | --- | --- | --- | --- |
| CNN | Conv1d | (Batch size, Input size, 85) | (Batch size, 16, 17) | 5 |
|  | Conv1d | (Batch size, 16, 17) | (Batch size, 32, 3) | 5 |
|  | BatchNorm1d | (Batch size, 32, 3) | (Batch size, 32, 3) |  |
|  | Conv1d | (Batch size, 32, 3) | (Batch size, 32, 1) | 3 |
|  | BatchNorm1d | (Batch size, 32, 1) | (Batch size, 32, 1) |  |
| LSTM | LSTM | (Batch size, 32) | (Batch size, 64) |  |
| Mean Estimator | Dropout | (Batch size, 64) | (Batch size, 64) |  |
|  | Linear | (Batch size, 64) | (Batch size, 128) |  |
|  | Linear | (Batch size, 128) | (Batch size, 128) |  |
|  | Linear | (Batch size, 128) | (Batch size, 100) |  |
| StD Estimartor | Dropout | (Batch size, 64) | (Batch size, 64) |  |
|  | Linear | (Batch size, 64) | (Batch size, 128) |  |
|  | Linear | (Batch size, 128) | (Batch size, 100) |  |
|  | Softplus | (Batch size, 100) | (Batch size, 100) |  |

For NCM+NCA cells.

| **Module** | **Layer** | **Input shape** | **Output shape** | **Kernel size** |
| --- | --- | --- | --- | --- |
| CNN | Conv1d | (Batch size, Input size, 25) | (Batch size, 16, 5) | 5 |
|  | Conv1d | (Batch size, 16, 5) | (Batch size, 32, 1) | 5 |
|  | BatchNorm1d | (Batch size, 32, 1) | (Batch size, 32, 1) |  |
| LSTM | LSTM | (Batch size, 32) | (Batch size, 64) |  |
| Mean Estimator | Dropout | (Batch size, 64) | (Batch size, 64) |  |
|  | Linear | (Batch size, 64) | (Batch size, 128) |  |
|  | Linear | (Batch size, 128) | (Batch size, 128) |  |
|  | Linear | (Batch size, 128) | (Batch size, 100) |  |
| StD Estimartor | Dropout | (Batch size, 64) | (Batch size, 64) |  |
|  | Linear | (Batch size, 64) | (Batch size, 128) |  |
|  | Linear | (Batch size, 128) | (Batch size, 100) |  |
|  | Softplus | (Batch size, 100) | (Batch size, 100) |  |

**Note S6: PyBaMM simulation**


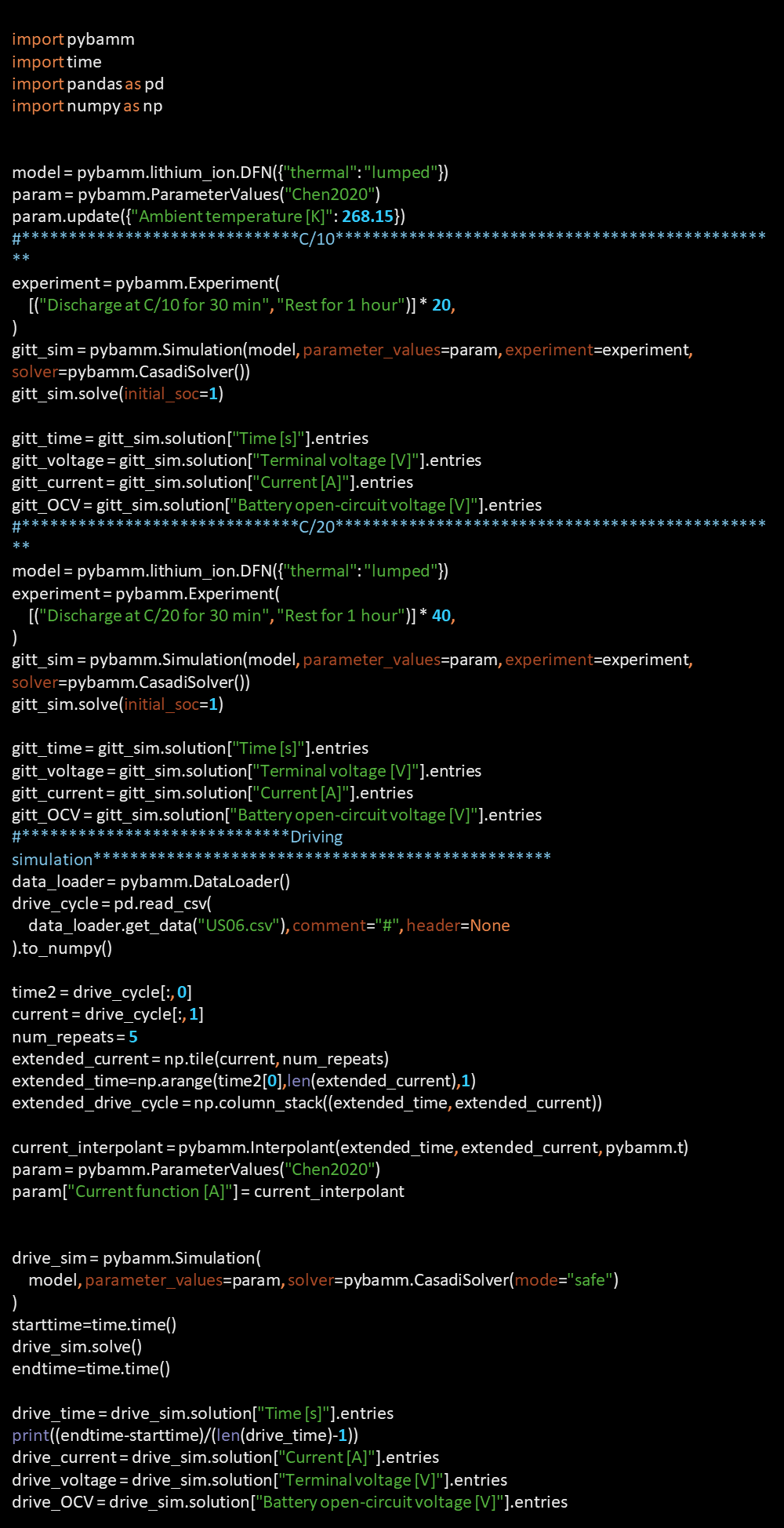

Supplement: Supplementary file 1 — Supporting Information [file ADVS-13-e06772-s001.docx]
